# Chronic *Toxoplasma* infection is associated with distinct alterations in the synaptic protein composition

**DOI:** 10.1186/s12974-018-1242-1

**Published:** 2018-08-01

**Authors:** Daniel Lang, Björn H. Schott, Marco van Ham, Lorena Morton, Leonora Kulikovskaja, Rodrigo Herrera-Molina, Rainer Pielot, Frank Klawonn, Dirk Montag, Lothar Jänsch, Eckart D. Gundelfinger, Karl Heinz Smalla, Ildiko Rita Dunay

**Affiliations:** 1 Institute of Inflammation and Neurodegeneration, Otto von Guericke University Magdeburg, Magdeburg, Germany; 2 Leibniz Institute for Neurobiology, Magdeburg, Germany; 3 Helmholtz Centre for Infection Research, Cellular Proteomics Group, Braunschweig, Germany; 4 Medical Faculty, Department of Neurology, Otto von Guericke University Magdeburg, Magdeburg, Germany; 5 Center for Behavioral Brain Sciences, Magdeburg, Germany; 6 Molecular Neurobiology, Medical Faculty, Otto von Guericke University Magdeburg, Magdeburg, Germany; 7 Centro Integrativo de Biología y Química Aplicada, Universidad Bernardo O’Higgins, Santiago, Chile; 8 Department of Computer Science, Ostfalia University of Applied Sciences, Wolfenbuettel, Germany

**Keywords:** *Toxoplasma gondii*, Chronic *Toxoplasma* infection, Synaptic proteins, Neuroinflammation

## Abstract

**Background:**

Chronic infection with the neurotropic parasite *Toxoplasma gondii* has been implicated in the risk for several neuropsychiatric disorders. The mechanisms, by which the parasite may alter neural function and behavior of the host, are not yet understood completely.

**Methods:**

Here, a novel proteomic approach using mass spectrometry was employed to investigate the alterations in synaptic protein composition in a murine model of chronic toxoplasmosis. In a candidate-based strategy, immunoblot analysis and immunohistochemistry were applied to investigate the expression levels of key synaptic proteins in glutamatergic signaling.

**Results:**

A comparison of the synaptosomal protein composition revealed distinct changes upon infection, with multiple proteins such as EAAT2, Shank3, AMPA receptor, and NMDA receptor subunits being downregulated, whereas inflammation-related proteins showed an upregulation. Treatment with the antiparasitic agent sulfadiazine strongly reduced tachyzoite levels and diminished neuroinflammatory mediators. However, in both conditions, a significant number of latent cysts persisted in the brain. Conversely, infection-related alterations of key synaptic protein levels could be partly reversed by the treatment.

**Conclusion:**

These results provide evidence for profound changes especially in synaptic protein composition in *T. gondii*-infected mice with a downregulation of pivotal components of glutamatergic neurotransmission. Our results suggest that the detected synaptic alterations are a consequence of the distinct neuroinflammatory milieu caused by the neurotropic parasite.

**Electronic supplementary material:**

The online version of this article (10.1186/s12974-018-1242-1) contains supplementary material, which is available to authorized users.

## Background

The seroprevalence of *Toxoplasma gondii* (*T*. *gondii*) reaches up to 50% in humans worldwide but varies considerably between different world regions [1, 2]. Transmission occurs predominantly via oral ingestion and typically results in latent infection of the host’s central nervous system (CNS) [2, 3]. The fast-replicating virulent tachyzoites can invade all nucleated cells in the brain, which evokes an efficient immune response by activation of resident glia and recruitment of peripheral immune cells [4–6]. Some parasites elude their elimination by invading neurons and transforming into slowly replicating bradyzoites protected by the cyst wall [7]. Intraneuronal cysts may persist lifelong, causing subclinical basal neuroinflammation [8]. No currently available antiparasitic drug is able to eliminate intracellular cysts, but the number of tachyzoites can be reduced by drugs such as the sulfonamide antibiotic sulfadiazine, one of the gold standards in clinical practice [9].

A remarkable feature of *T*. *gondii* is the parasite’s ability to alter the host’s behavior. Most prominently, infected rodents lose their natural aversion towards feline predators, which are *T*. *gondii*’s definite host species [10–13]. While some studies have suggested this behavioral change to be highly specific, others have reported more general sensorimotor deficits [14–16]. In humans, most studies on behavioral sequelae of latent *T*. *gondii* infection have implicated *T*. *gondii* seropositivity in the risk for several neuropsychiatric conditions, including depression, disorders associated with autoagression, and psychotic disorders, particularly schizophrenia [17, 18]. Possible associations of chronic *T*. *gondii* infection with subclinical behavioral alterations are less well studied and currently discussed [19]; however, a number of psychomotor and cognitive functions have been suggested to be altered in *T*. *gondii*-seropositive individuals [20, 21].

One promising candidate mechanism, by which *T*. *gondii* could exert such diverse effects on behavioral phenotypes, might be interference with the synapse function. Synaptic transmission underlies highly sophisticated regulation by protein networks assembled at the presynaptic site of neurotransmitter release and the postsynaptic apparatus for neurotransmitter reception. The concept of synapses has evolved over the past two decades from a simple neuronal communication connection device consisting of the pre- and postsynaptic compartments into a highly complex structure. Various findings [22–24] have highlighted the involvement of glial cell protrusions in the synaptic function leading to the now widely accepted concept of a tripartite synapse [25–28]. More recently, the extracellular matrix (ECM) was proposed as a critical fourth element in this functional complex to form a tetrapartite synapse [29, 30]. This complexity is also reflected in biochemical synaptic protein preparations (synaptosomes) that contain, in addition to typical pre- and postsynaptic components, ECM elements as well as proteins of astrocytic and microglial origin (e.g., www.SynProt.de; [31]).

Over the past two decades, increasing evidence has been obtained for a modulatory role of neuroinflammation in synaptic plasticity and in ultimately learning and memory processes [32, 33]. Chronic *T*. *gondii* infection is associated with a neuroinflammatory response, which is not restricted to the areas surrounding the cysts, but rather exerts widespread effects throughout the CNS. The Th1 cell-mediated immune response triggered by *T*. *gondii* [34] is associated with activation of resident and recruited immune cells, resulting in the release of several cytokines and chemokines, such as interferon gamma (IFN-γ), interleukin-12 (IL-12), interleukin-1 (IL-1), interleukin-6 (IL-6), and the tumor necrosis factor (TNF) [5, 6, 35–37]. Recent studies have revealed substantial effects of distinct cytokines on modulating different brain functions [33, 38]. At the clinical level, the pro-inflammatory cytokine IFN-γ, a key molecule in *T*. *gondii*-induced neuroinflammation [39], has been implicated in major depression, obsessive-compulsive disorder, and schizophrenia [40], and similar associations have been observed for IL-1, IL-6, IL-12, and TNF. Pro-inflammatory cytokines modulate neuronal function, with optimal levels being putatively neuroprotective, while higher levels exert neurotoxic effects [32]. Within this framework, IFN-γ plays a neuroprotective role by inducing transforming growth factor beta-1 (TGFβ-1), which in turn prevents the production of neurotoxic nitric oxide (NO) [41]. Accordingly, neurodegeneration in *T*. *gondii*-infected brains is rather limited [8, 42]. Nevertheless, *T*. *gondii*-activated cytokines disrupt synaptic signaling. As outlined by Haroon and colleagues [43], IFN-γ, TNF, IL-1, and IL-6 interfere with plasticity-related synaptic signaling pathways, thereby influencing signaling related to excitotoxicity and oxidative stress.

Mahmoudvand and colleagues further reported that increased expression of TNF, IL-1β, and IL-6 in astrocytes of *T*. *gondii*-infected animals [44] was associated with increased pain sensitivity. Considering that somatosensory, including pain, pathways depend on glutamatergic signaling, it is plausible to assume that infection may also affect glutamatergic signaling. Recently, Klein et al. [33] have indeed emphasized the importance of glutamatergic excitatory neurotransmission in the inflammation-related modulation of hippocampal learning and memory (IL-1β, TNF, C-X-C motif chemokine 10) and neurogenesis (IFN-γ, TNF, IL-1ßγ). Accordingly, a recent study has revealed changes in glutamatergic signaling in mice with chronic *T*. *gondii* infection [45]. Most notably, the excitatory amino acid transporter 2 (EAAT2; also known as glutamate transporter 1, GLT-1), which mediates the rapid uptake of released glutamate into perisynaptic astrocytes [46], was downregulated in infected mice. Importantly in this context, application of the β-lactam antibiotic ceftriaxone, which has anti-inflammatory but no antiparasitic properties, resulted in a partial restoration of downregulated EAAT2/GLT-1 in infected mice, while levels of glutamine synthethase (GS) remained unchanged. Impaired inhibitory GABAergic synaptic transmission following *T*. *gondii* infection has also been observed [47]. Specifically, the distribution of the GABA-synthesizing enzyme glutamate decarboxylase 67 (GAD67) was more diffusely distributed in infected than in control animals. Also, infected mice developed spontaneous seizures, potentially reflecting a GABAergic deficit at the system level.

We have previously demonstrated alterations in microscopic neuroanatomy within the brains of chronically *T*. *gondii*-infected mice [8]. At the level of microstructure, we observed infection-associated increases in ventricle size, occasional lesions throughout the brain, and also reduced fiber density in the cortical areas. Those changes were further associated with a loss in structural complexity of axons and dendrites at the ultrastructural level. Markers for pre- and postsynaptic function, synaptophysin and postsynaptic density protein 95 (PSD-95/Dlg4) were reduced in the neocortex and hippocampus of infected mice. Given this downregulation of two important synaptic proteins in infected mice and the previous results concerning alterations in both glutamatergic and GABAergic neurotransmission, we hypothesized that *T*. *gondii*-related changes in synaptic biochemistry would most likely be extensive. Compatibly, a very recent study could demonstrate major changes in the transcriptomes of *T*. *gondii*-infected compared to uninfected primary cultures from human neural stem cells [48]. Our present study employed a combined proteomic- and candidate-based biochemical approach to elucidate how *T*. *gondii* infection alters synaptic protein composition from isolated synaptosomal fractions. Finally, we treated the infected mice with the antiparasitic drug sulfadiazine to diminish tachyzoite numbers and associated neuroinflammation and to discern latent *Toxoplasma* infection-mediated synaptic alterations.

## Methods

### Infections with *T*. *gondii* and sulfadiazine treatment

For infections, *T*. *gondii* cysts of the ME49 strain were used as described previously [42]. Parasites were harvested from the brains of NMRI mice infected 6 to 10 months earlier with *T*. *gondii* cysts. Control (non-infected) mice were mock-infected with sterile PBS. Experiments were conducted 5 weeks post-infection. Subgroups of both mock-infected and *T*. *gondii*-infected mice received sulfadiazine treatment via drinking water (400 mg/l) ad libitum cursive from day 10 until week 5 after infection.

### Tissue processing and synaptosome preparation

Five weeks after infection, the mice were anesthetized with isoflurane (PP Pharma, Germany) and decapitated. The forebrains and midbrains were harvested, and the regions of interest (neocortex, hippocampus, and subcortical areas) were dissected from both hemispheres for proteomics and immunoblot analysis. Subcellular fractionation was performed by differential centrifugation and sucrose density gradient ultracentrifugation as described previously [49] (for details, see Additional file 1).

### Proteomics

Four independent infected and four independent non-infected samples from three experiments were used for the proteomics studies in total. In all studies, synaptosomal fractions were digested, and peptides were labelled with isobaric tags for relative and absolute quantitation (iTRAQ)-labeled and combined for comparative quantification by highly accurate Fourier transform mass spectrometry.

#### Protein digest

Synaptosomal proteins were digested with trypsin (Trypsin Gold, MS grade, Promega Germany, Mannheim) in 50 mM triethylammonium bicarbonate (TEAB) containing 10% acetonitrile (ACN). A protein/protease ratio of no more than 50:1 was applied, and digestion was performed at 37 °C overnight. Peptides were vacuum-dried, resolved in 0.2% trifluoroacetic acid (TFA) in water, desalted on self-packed LiChroprep RP18 (10 μl, Merck), eluted in 0.2% TFA and in 60% ACN, and dried again.

#### iTRAQ labeling and SCX chromatography

iTRAQ labeling was performed according to the manufacturer’s protocol (Applied Biosystems), and the following iTRAQ reporter combinations were used: *T*. *gondii*-infected mice 114(/115) and non-infected mice 116(/117). Labeled peptide mixtures were subfractionated as described previously [50] (more details in Additional file 1).

#### LC-MS/MS and data analyses

LC-MS/MS analyses were performed on a DionexUltiMate 3000 n-RSLC system connected to an Orbitrap Fusion mass spectrometer (Thermo Scientific). Peptides were loaded onto a C_18_ pre-column (3 μm, Acclaim, 75 μm × 20 mm, Dionex) and washed for 3 min at a flow rate of 6 μl/min. Subsequently, peptides were separated on a C_18_ analytical column (2 μm, Acclaim PepMap RSLC, 75 μm × 25 cm, Dionex) at 200 μl/min via a linear 120-min gradient with ultra performance liquid chromatography (UPLC) buffer A (0.1% FA in water) and 25% UPLC buffer B (0.1% formic acid in ACN), followed by a 60-min gradient from 25 to 50% of buffer B. The LC system was operated with Chromeleon Software (version 6.8, Dionex) embedded in Xcalibur software (version 2.1, Thermo Scientific). The effluent from the column was electro-sprayed (Pico Tip Emitter Needles, New Objectives) into the mass spectrometer. The mass spectrometer was controlled by Xcalibur software and operated in the data-dependent mode allowing for automatic selection of a maximum of ten doubly and triply charged peptides and their subsequent fragmentation. A dynamic exclusion allowed up to one repetition after every 12 s. Peptide fragmentation was carried out using High Collision Dissociation settings optimized for iTRAQ-labeled peptides. MS/MS raw data files were processed via Proteome Discoverer 1.3.0.339 mediated searches against UniProtKB/Swiss-Prot protein database (release 2013_01, with 538,849 entries; taxonomy *Musmusculus* with 16,589 entries) on a Mascot server (V. 2.4, Matrix Science). The following search parameters were used: enzyme, trypsin; maximum missed cleavages, 1; fixed modifications, iTRAQ 4-plex (K), iTRAQ (N terminus), Methylthio (C), oxidation (M); peptide tolerance, 5 ppm; MS/MS tolerance, 100 mmu. In order to identify proteins significantly regulated by *T*. *gondii* infection, we computed the binary logarithm of the ratio between the protein amounts detected in infected versus uninfected animals for each protein identified (log_2_ [prot_inf_/prot_con_], hence termed *regulation factor*). Quantile-based inference statistical testing was employed to statistically verify that these regulation factors did not result from merely random variation (see Additional file 1 for details).

#### Meta-analysis of proteomics results

Statistical analysis of the functional annotations characterized by the regulated proteins was performed using three different tools: First, a single enrichment analysis was performed using the annotation databases Gene Ontology (http://geneontology.org/) and KEGG (http://www.genome.jp/kegg/), in conjunction with in-house scripts. The scripts use three different random protein lists as control; the statistical significance of enriched biological processes, molecular functions, and pathways is calculated by Fisher’s exact test, and the *p* values are corrected for a false discovery rate (FDR) of 0.05 using the Benjamini-Hochberg procedure [51]. If an annotation was significantly enriched in all comparisons to the random protein lists, this annotation is defined finally as enriched. The web-based tools GeneCodis (http://genecodis.cnb.csic.es/) and DAVID (https://david.ncifcrf.gov/) were employed for a modular enrichment analysis, using the default parameters in both tools. The statistical significance of groups of functional annotations was calculated by comparison to the whole proteome as a control. The *p* values were FDR-corrected using the Benjamini-Hochberg method. Furthermore, QIAGEN’s Ingenuity® Pathway Analysis (IPA®, QIAGEN Redwood City, www.qiagen.com/ingenuity).”<http://www.qiagen.com/ingenuity)>“) was also used for extended functional analyses of infection-associated proteome changes to identify significant canonical pathways, diseases and biofunctions, molecular and cellular functions or relations to physiological system development with *p* < 0.05, and sufficiently large cluster sizes.

### Candidate-based investigations

All analyses were performed in two to three independent experiments with *n* = 4 to 8 animals per group.

#### Protein concentration determination

Bicinchoninic Acid (BCA) protein assay kit (Interchim, Montluçon, France) was used according to the manufacturer’s protocol to achieve a protein concentration of 2 μg/μl. The samples were incubated either at 95 °C for 5 min or alternatively at 37 °C for 1 hour. Additionally, equal sample loading on the gels was further verified by Coomassie brilliant blue staining.

#### SDS-PAGE and immunoblot analysis

##### SDS-PAGE

Samples of proteins (30 μg) and Precision Plus Protein™ Dual Color Standard (Bio-Rad, USA) were running on a 5–20% gradient polyacrylamide gel at a constant current of 9 mA. Gels were used for either Coomassie staining or immunoblotting.

##### Coomassie staining

The gels were stained with Imperial™ Protein Stain (Thermo Scientific, USA) overnight and destaining for 3 h with de-ionized water. After destaining, optical densities were evaluated (Quantity One® 1-D analysis software, Bio-Rad, USA).

##### Immunoblotting

Proteins were electro-transferred onto nitrocellulose membranes (Protran® BA85, Whatman®, Dassel, Germany) at 4 °C for 1.5 or 2.5 h for improved transfer of larger proteins (Mr > 150 kDa) and at 200 mA (TE22 tank blotting unit, Hoefer, USA).

##### Immunoblot detection

Membranes were blocked for 1 h at RT with 5% skimmed milk powder in TBS-T. Blots were then incubated at 4 °C overnight with primary antibodies diluted in TBS-A. The membranes were submerged in peroxidase-labeled secondary antibodies in TBS-T/5% skimmed milk powder for 1.5 h at RT. The following primary antibodies were applied: rabbit anti-EAAT2 (1:1000, K.O. controlled antibody), guinea pig anti-Shank3 (1:500), rabbit anti-GABAAα1 (1:1000), rabbit anti-Parvalbumin (1:1000), mouse anti-GFAP (1:1000), rabbit anti-GluN1 (1:1000), rabbit anti-GluN2B (1:1000), rabbit anti-GluA1 (1:1000), and rabbit anti-GluA2 (1:1000) were purchased from Synaptic Systems, Göttingen, Germany. For secondary HRP-conjugated antibodies, goat anti-rabbit (1:10000, Jackson ImmunoResearch, Westgrove, PA, USA), rabbit anti-guinea pig (1:9000, Agilent, Santa Clara, CA, USA), and rabbit anti-mouse (1:10000, Merck, Darmstadt, Germany) were applied. For detection, membranes were incubated with Pierce® ECL Immunoblotting Substrate (Thermo Scientific, USA) for 5 min. For semi-quantitative studies, the chemiluminescence signals were captured for 1 to 2 min using an INTAS Imager (INTAS Science Imaging Instruments, Göttingen, Germany). Single immunostained band optical densities were evaluated using open source software Fiji version 1.0 and finally normalized to the mean value of all band densities on a single blot. For immunoblots related to sulfadiazine experiments, bands were also normalized to Tubulin signal.

### Immunofluorescence

Mice were transcardially perfused with PBS and 4% paraformaldehyde before organ collection (Affymetrix, USA). Hippocampal (Bregma − 2.06 to − 1.70 mm, interaural 1.74 to 2.10 mm) and cortical (Bregma 0.38 to 0.50 mm, interaural 4.18 to 4.30 mm) sections were blocked in normal horse serum (HS) (Thermo Scientific, USA). The slices were incubated with primary antibodies in horse serum and 0.3% Triton-X-100 (Roth, Karlsruhe, Germany) in PBS at 4 °C for 40–44 h. The sections were incubated with either AlexaFluor 488 or Cy3 or Cy5 conjugated secondary antibodies in the same buffer for 4 h. For additional staining, guinea pig anti-MAP2 (1:2000) (Synaptic Systems, Göttingen, Germany), mouse anti-MAP2 (1:1000) from Sigma-Aldrich (St. Louis, USA), and DAPI (1 mg/ml; Sigma-Aldrich, USA) were applied. Corresponding donkey secondary antibodies (AlexaFluor 488, Cy3 or Cy5 conjugated, 1:1000) were purchased from Jackson ImmunoResearch Europe (Suffolk, UK). Images from Mowiol 40-88 (Sigma-Aldrich, USA)-mounted slices were acquired with a Leica TCS SP5 confocal microscope, while we tried to prevent signal saturation. Overview images of the sections were compiled from image series of tiles, captured with a × 10 objective. Tiles with an overlap of 10% for *x* and *y* from each brain were composed to single images with Leica software LAS AF.

#### Quantification of immunohistochemical stainings

Composed images were quantified with ImageJ-based Fiji software (ImageJ-Version 2.0.0-rc-56/1.5j). Integrated density (ID) values for each ROI (Additional file 2) were calculated as relative values (ID_Rel_ = ID_ROI_/ID_Ref_). The hypothalamus (HTH) was used as the reference area (Ref). For each protein staining, three cortical sections for uninfected and infected mice were selected. In order to avoid quantification of false-positive signals, we excluded the areas of artifacts with saturated intensity when the individual ROIs were selected.

### Cyst burden count

The brain samples were collected on day 10, 20, or 35. One hemisphere was stored in RNA later (Quiagen) for further processing via qRT-PCR, and another hemisphere was used to determine cyst numbers, as previously described [42]. After homogenization in 1 ml PBS, 10 μl (four times) of brain suspensions was screened via light microscopy (× 20 magnification). Three to four individuals were investigating the brain homogenates (blinded), to determine the estimated total cyst count per brain.

### qRT-PCR from brain homogenates

For qRT-PCR analysis, TaqMan® Assay with RNA-to-C_T_™ 1-Step Kit (Life Technologies, Germany) in a LightCycler® 96 (Roche, Germany) was used. TaqMan® Gene Expression Assays (Life Technologies, Germany) for hypoxanthine-guanine phosphoribosyltransferase (HPRT; Mm01545399_m1), IFN-γ (Mm00801778_m1), TNF (Mm00443258_m1), IL-10 (Mm00439616_m1), IL-12a (Mm00434165_m1), IL-6 (Mm00446190_m1), IL-1β (Mm00434228_m1), and SAG1 (tachyzoites).

## Results

### Proteomics

#### Infection-related alterations of protein composition in synaptosomes

**Fig. 1:**
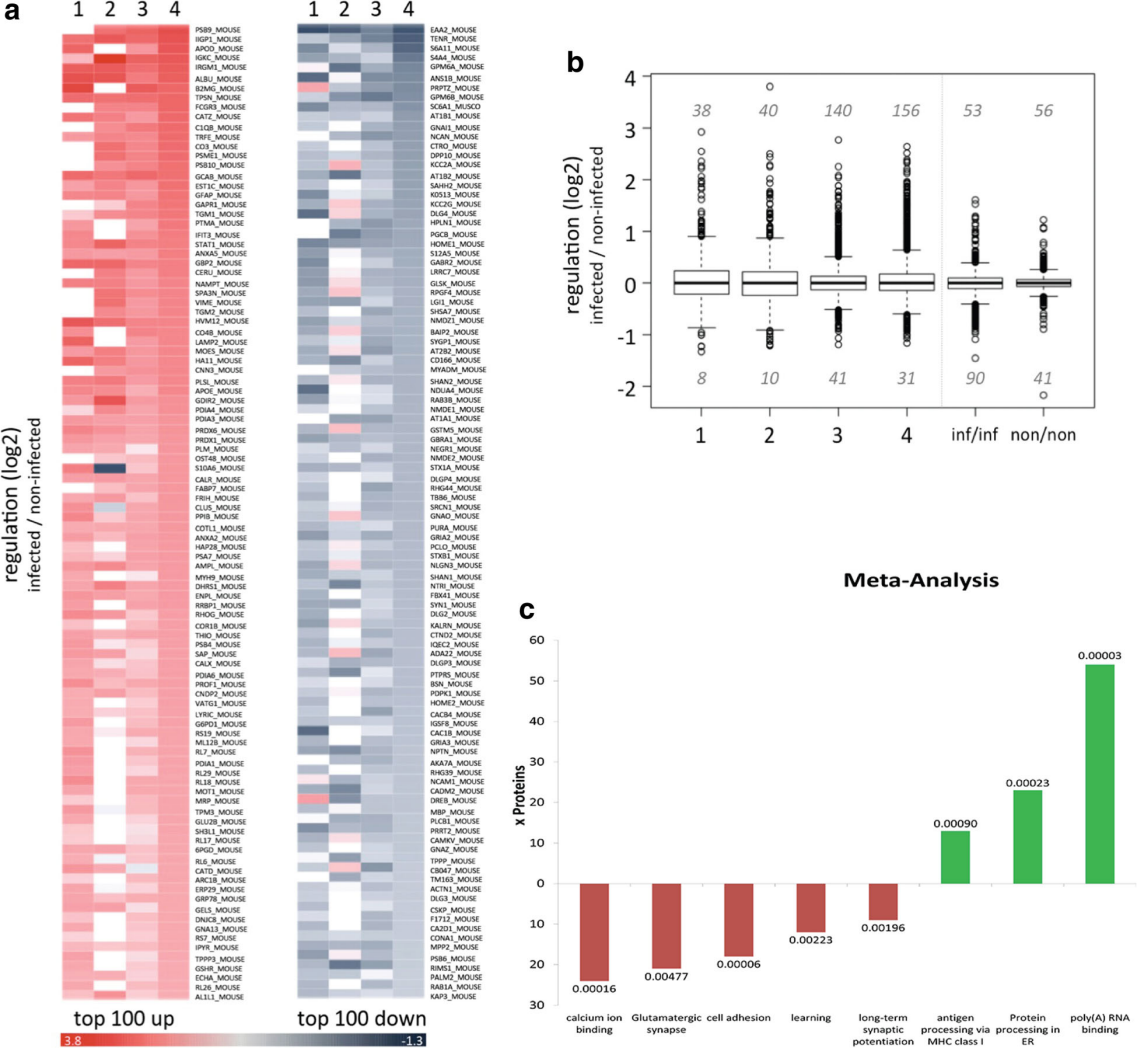
Proteomic analysis of synaptosomes from *Toxoplasma*-infected mice. Synaptosomes from infected and non-infected mice were isolated, proteins were digested, and peptides were iTRAQ labeled and analyzed using mass spectrometry. **a** Heatmaps showing relative protein abundances (log2) of the top 100 significantly upregulated (left) and downregulated (right) proteins in synaptosomes after *T*. *gondii* infection in four separate sample pairs. Color codes are indicated. **b** Boxplots showing regulations (log_2_) of protein abundances from infected and non-infected samples from four separate sample pairs (1–4) and from infected (inf/inf) and non-infected (non/non) synaptosomal fractions from one experiment. **c** Single enrichment analysis of the up- and downregulated proteins. The *y*-axis denotes the number of proteins, which were assigned to the KEGG- or GO annotation; upregulated functions or pathways are depicted in green; downregulated functions or pathways in red. Only significant enriched annotations from KEGG and from annotations related to “biological process” and “molecular function” from Gene Ontology database are depicted. The values on top of the bars denote the *p* values. The GO annotation “antigen processing via MHC class I” stands for “antigen processing and presentation of exogenous peptide antigen via MHC class I, TAP-dependent.” The KEGG annotation “protein processing in ER” stands for “protein processing in endoplasmic reticulum”

Synaptic differences between *T*. *gondii* infected and non-infected brains were analyzed at the level of synaptosomal protein abundances. Database searches were restricted to high-quality MS data and identified a total of 3062 proteins in synaptosomes from infected and non-infected mice (Additional file 3). Among these, 694 proteins were identified in all experiments, and 1225 proteins were detected in at least three sample sets, indicative of reproducible and robust information on protein abundances (Additional file 4). Overall, we observed a prominent upregulation of synaptosomal protein abundances in the synaptosomes of infected animals. Among the regulated proteins, approximately 80% were more abundant in synaptosomal fractions from infected mice, while 20% were less abundant (Fig. 1b).

We performed also four-plex iTRAQ labeling (samples 3 and 4) using synaptosomes isolated from two infected and two non-infected mice, allowing us to not only compare protein abundances between groups (infected vs. non-infected mice) but also within the two groups. When comparing non-infected mice, fewer synaptosomal proteins showed (most likely incidental) differences in abundances. Upon infection, the number of proteins that differed in their synaptosomal abundances increased; however, it was still substantially lower than in the comparison of infected and non-infected animals (Fig. 1b; documented by the width of whisker plots). Thus, our approach comprising the synaptosome isolation protocol and subsequent mass spectrometry was sufficiently powerful for the comparative analyses of infected versus non-infected mice.

Statistical analyses revealed that, of all 3062 proteins found, a total of 567 proteins exhibited significantly different abundances in the synaptosomal fractions of infected compared to non-infected mice (Additional file 3). When analyzing proteins that were found in at least three of the four separate sample sets, we identified 292 proteins displaying significantly different abundances as a function of infection status (Additional file 3).

Relative changes of abundances of these 292 proteins are displayed as heat maps; Fig. 1a shows the top 100 up- or downregulated proteins; and Additional file 5 shows all 292 significantly regulated proteins. The most prominent downregulation was observed for EAAT2. Infection-related downregulation was also observed for several synaptic proteins previously identified in genetic studies of neuropsychiatric disorders like bipolar disorder (neurocan [52]), major depression (Homer-1 [53]; Piccolo [54]), or autism (Shank2 [55]).

#### Meta-analyses of proteomics data

To elucidate the impact of the infection-related synaptosomal proteome changes as determined from our MS data at the level of functional systems, the list of significantly regulated proteins was split into upregulated and downregulated proteins. Single enrichment analysis of the upregulated proteins showed a pronounced upregulation of proteins related to “poly(A)-RNA binding” and further revealed a significant overrepresentation of annotations linked to immune responses of a cell, such as “antigen processing and presentation of exogenous peptide antigen via MHC class I, TAP-dependent” (Fig. 1c). The same analysis performed on downregulated proteins showed a notable overrepresentation of proteins involved in neuronal functions, including “glutamatergic synapse” and “calcium binding” (Fig. 1c). This pattern was further supported by modular enrichment analysis using DAVID and GeneCodis; The upregulated proteins showed an overrepresentation of immune response functions like “protein processing in ER”, “antigen processing” (GeneCodis), “antigen processing and presentation,” and “immune response” (DAVID). Downregulated proteins were frequently related to neuronal function, including, e.g., “receptor activity,” “synaptic transmission” (GeneCodis), “ion channel activity,” or “ion transport” (DAVID). The complete results from DAVID and KEGG are provided as Additional file 1.

### Candidate-based studies

As proteomics indicated downregulation of both glutamatergic and GABAergic synapse components in *T*. *gondii* infected mice (Fig. 1c, Additional file 6, and Additional file 7), we sought to further substantiate our observations by technically independent, candidate-based immunoblot analyses of principal indicators of glutamatergic and GABAergic synapse function. Markers of glutamatergic synapse function included the AMPA receptor subunits GluA1 and GluA2, the NDMA receptor subunits GluN1 and GluN2B, and the glutamate transporter EAAT2 and the postsynaptic scaffolding protein Shank3. The GABA_A_ receptor α1 subunit was chosen as a marker of GABAergic function. The glial fibrillary acidic protein (GFAP), which is highly expressed in activated astrocytes, served as an indicator of neuroinflammation and resulting astrogliosis [56, 57].

#### Immunoblot analysis of synaptic components

**Fig. 2:**
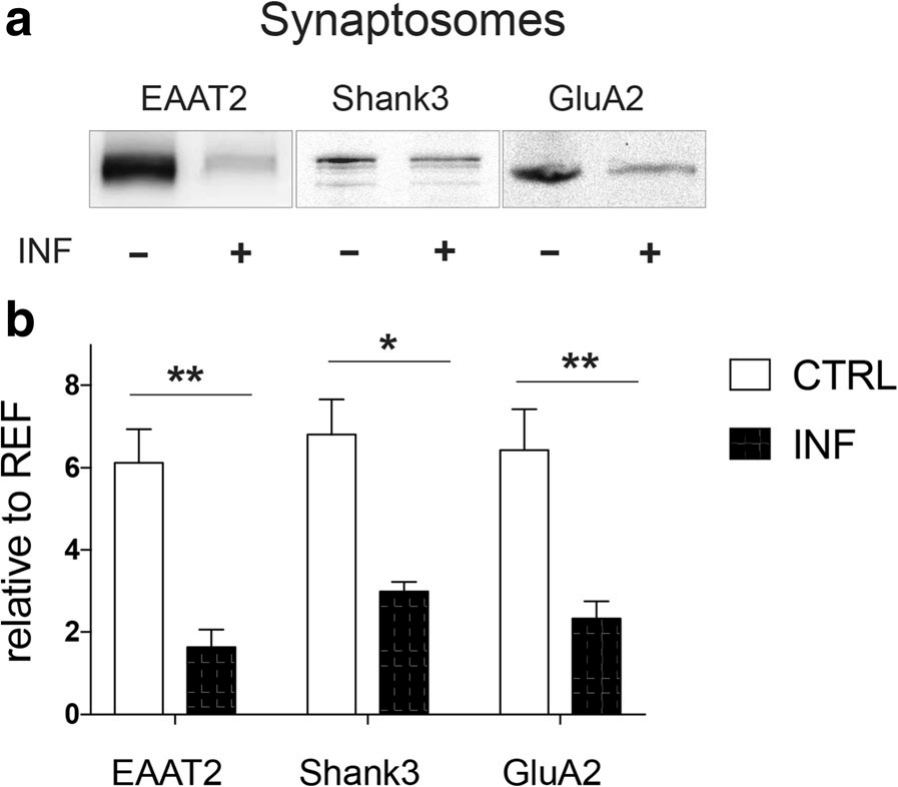
Altered synaptic composition upon chronic *Toxoplasma* infection. Immunoblots (**a**) and statistica analysis (**b**) from the cortical synaptosomes revealed significant downregulation of EAAT2, Shank3, and GluA2 in infected tissue (INF+). Control mice (CTRL) *n* = 8, infected mice (INF) *n* = 4. Displayed results are mean values ± SEM. **p < 0.01, *p < 0.05

Immunoblot results were essentially compatible with the overall downregulation of proteins related to synaptic function observed in the proteomics experiments (Fig. 1a, Additional file 5). Immunoblot analysis of synaptosomal fractions (Fig. 2) from infected (*n* = 4) and non-infected (*n* = 8) animals revealed for EAAT2, GluA2, Shank3, and GABA_A_R α1 a highly significant main effect of infection status (*F*_1,10_ = 12.93; *p* = 0.005, two-way repeated measures ANOVA with protein as a within-subject factor and infection status as a between-subject factor) while no significant effect of protein or interaction was observed (all *p* > 0.804). Post hoc two-sample *t* tests further showed that this effect was driven by glutamatergic system components, with significant infection-related reduction of EAAT2, GluA2, and Shank3 (all *t* > 3.00; all *p* < 0.013; unequal variances assumed, see Fig. 2b), while the amount of GABA_A_R α1-subunits was not significantly different between infected and uninfected animals (*p* > 0.13; see Additional file 8).

#### Global alteration of synaptic protein levels in different brain regions

To investigate the infection-related regulation of synaptic proteins at a global level, we performed immunoblot analyses of the neocortex and hippocampus homogenates. Furthermore, we analyzed the distribution of selected proteins by immunofluorescence staining in coronal brain sections.

#### Downregulation of glutamatergic synapse components in the neocortex and hippocampus

**Fig. 3:**
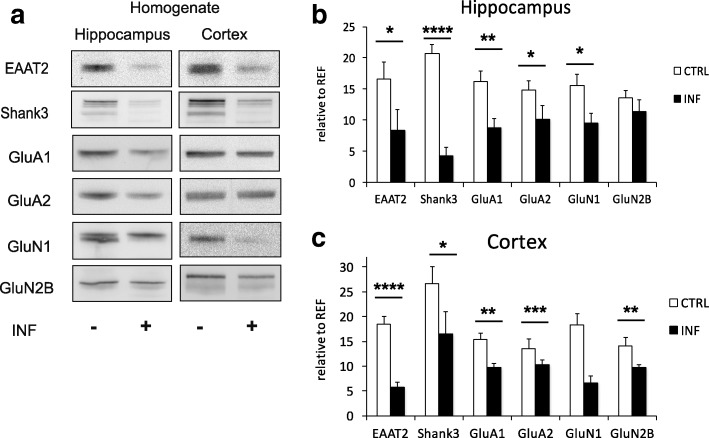
Chronic *Toxoplasma* infection reduces synaptic protein expression in the whole brain. **a** Immunoblot analysis from brain homogenate in the neocortex and hippocampus for glutamatergic synaptic proteins. Relative protein levels of glutamate transporter EAAT2, postsynaptic scaffolding protein Shank3, and glutamate receptors GluA1, GluA2, GluN1, and GluN2B were quantified. **b** revealed downregulation of EAAT2, Shank3, GluA1, GluA2, and GluN1 in the hippocampus. **c** Data also revealed downregulation of EAAT2, Shank3, GluA1, GluN1, and GluN2B in the cortex of homogenate. Control mice (CTRL) *n* = 8, infected mice (INF) *n* = 8. Displayed results are mean values ± SEM. ****p < 0.00001, ***p < 0.001, **p < 0.01, *p < 0.05

Immunoblot analysis of homogenized brain tissue using antibodies against glutamatergic synapse components (EAAT2, Shank3, and the receptor subunits GluA1, GluA2, GluN1, GluN2B) revealed an overall downregulation of major synaptic proteins in the neocortex and hippocampus of infected compared to non-infected mice (Fig. 3a–c). In the cortex (Fig. 3c), a two-way ANOVA for repeated measures with protein as a within-subject factor, infection status as a between-subject factor, and experiment as a covariate (reflecting data from two independent experiments) revealed a main effect of infection status (*F*_1,13_ = 39.90; *p* < 0.001). There was also a main effect of protein, reflecting the different expression levels of the proteins, irrespectively of infection (*F*_2.8,36.6_ = 15.12; *p* < 0.001). Post hoc two-sample *t* tests suggested that this effect was attributable to EAAT2 (*t*_12.6_ = 6.99; *p* = 0.001), GluA1 (*t*_12.7_ = 3.57; *p* = 0.004; unequal variances assumed), GluN1 (*t*_12.0_ = 4.35; *p* = 0.001), and GluN2B (*t*_9.3_ = 4.35; *p* = 0.001), while only a trend-wise downregulation was observed for Shank3 (*t*_12.4_ = 1.85; *p* = 0.044, one-tailed). No significant downregulation in the cortical homogenates was observed for GluA2 (*t*_11.1_ = 1.40; *p* = 0.188).

A similar downregulation of glutamatergic synapse components was observed in the hippocampal homogenates (Fig. 3b) from infected animals (main effect of infection status: *F*_1,13_ = 30.84; *p* < 0.001; interaction protein by infection status: *F*_3.1,40.6_ = 3.23; *p* = 0.031, Greenhouse-Geisser correction applied; two-way repeated measures ANOVA with protein as a within-subject factor, infection status as a between-subject factor, and experiment 1 vs. 2 as a covariate). Post hoc two-sample *t* tests further revealed that the downregulation was significant for Shank3 (*t*_13.9_ = 8.47; *p* < 0.001), GluA1 (*t*_13.8_ = 3.37; *p* = 0.005), and GluN1 (*t*_13,8_ = 2.43; *p* = 0.029). In the homogenates of hippocampal probes, we further observed a trend-wise downregulation of EAAT2 (*t*_13,4_ = 1.90; *p* = 0.039, one-tailed), while the *t* test-based comparisons failed to reach significance for GluA2 (*p* = 0.101) and GluN2B (*p* = 0.326).

#### Minor alterations in the GABAergic system

*t* test-based comparisons of our immunoblot analyses of the GABA_A_ receptor α1 subunit (Additional file 8), as an exemplary protein of interest, revealed only a trend-wise infection-related difference in the neocortical (*t*_6_ = 2.14; *p* = 0.076), but not in the hippocampal homogenates (*p* > 0.600).

#### Functional neuroanatomy of changes in synaptic protein abundance

**Fig. 4:**
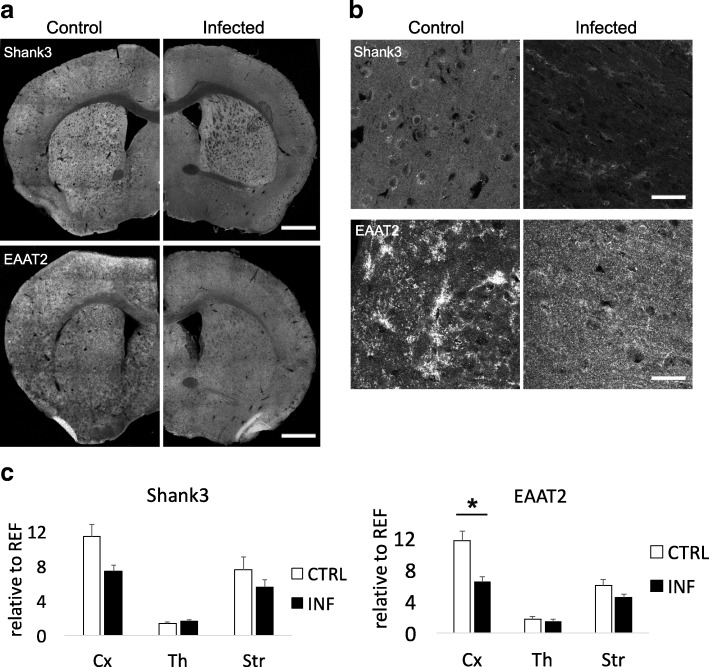
Glutamatergic signaling is affected upon chronic *Toxoplasma* infection. The coronal sections of *Toxoplasma gondii*-infected (INF) and control animals (CTRL), stained with markers of glutamatergic synapses, the postsynaptic scaffolding protein Shank3 and the glutamate transporter EAAT2, with focus on the specific cortical and subcortical areas (neocortex, thalamus, striatum). **a** Composed tile scan images with × 10 objective of whole brain and × 63 objective of somatosensory cortical areas (**b**). Integrated density (**c**) from each quantified area was normalized to hypothalamic reference area (see Additional file 2), indicating decreased cortical staining for EAAT2. Control mice (CTRL) *n* = 3, infected mice (INF) *n* = 3. Scale bars (**a**) 1 mm, scale bars (**b**) 40 μm. Displayed results are mean values ± SEM. *p < 0.05

To characterize the functional neuroanatomy underlying the observed synaptic protein downregulation, immunofluorescence (IF) staining was conducted in the coronal brain sections of infected and control animals (Fig. 4a). Integrated densities from each region of interest (ROI; neocortex, striatum, and thalamus) were normalized to the hypothalamus as the reference region (see Additional file 2) (Fig. 4c) In the neocortex, but not in the subcortical regions, markers of glutamatergic synapses (Shank3, EAAT2) showed consistently reduced staining intensity. A three-way ANOVA for repeated measures with protein (EAAT2, Shank3) and ROI (cortex, striatum, thalamus) as within-subject factors and infection status as between-subject factor showed a significant structure by infection interaction (*F*_2,5.6_ = 6.64; *p* = 0.039), and post hoc two-sample *t* tests further revealed a significant infection-related reduction of EAAT2 (*t*_3.0_ = 4.08; *p* = 0.027). We also found a trend-wise reduction of Shank3 (*t*_2.9_ = 2.56; *p* = 0.085, two-tailed) in the cortex. There was, on the other hand, no significant regulation of either Shank3 or EAAT2 in the subcortical areas (all *p* > 0.125).

With respect to GABA_A_ α1 (Additional file 8) as a marker for GABAergic neurotransmission, an ANOVA for repeated measures with region as a within-subject factor and infection status as a between-subject factor revealed no effect in the infection status or interaction in either the cortical or subcortical areas (neocortex, thalamus, striatum; all *p* > 0.155).

### Effects of sulfadiazine treatment

#### Synaptosomal protein alterations after sulfadiazine treatment

**Fig. 5:**
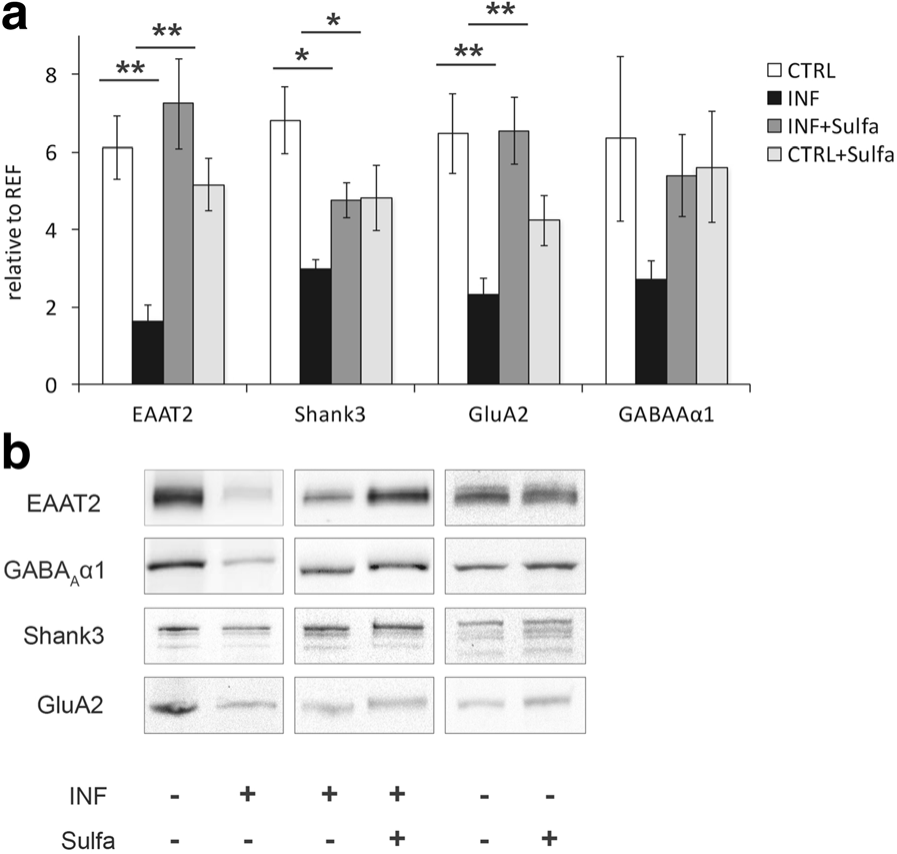
Synaptic protein levels after sulfadiazine treatment. **a** Comparison of cortical synaptosomes from untreated control mice (INF−), toxo-infected (INF+), toxo-infected mice treated with sulfadiazine (INF+ and Sulfa+), and control mice with sulfadiazine treatment (INF− and Sulfa+) via immunoblotting. Protein levels from all selected synaptic marker, EAAT2, GABA_A_α1, Shank3, and GluA2 were upregulated after treatment with sulfadiazine. Control staining with synaptosomes from untreated mice and mice treated with sulfadiazine showed no significant differences. **b** According blots to the graph from **a**, treatment for each blot is indicated below: *Toxoplasma* positive (INF+) or negative (INF−) tissue, as well as sulfadiazine treatment (Sulfa+) are labeled accordingly. (Control mice (CTRL) *n* = 8, infected mice (INF) *n* = 4, infected mice with sulfadiazine (INF + Sulfa) *n* = 4, and control mice with sulfadiazine treatment (CTRL + Sulfa) *n* = 4). Displayed results are mean values ± SEM. **p < 0.01, *p < 0.05

We next treated *T*. *gondii*-infected mice with the sulfonamide antibiotic sulfadiazine and compared immunoblots of cortical synaptosomal fractions from treated mice to untreated infected and control mice. Sulfadiazine-treated infected mice showed upregulation of synaptic marker proteins when compared to untreated infected animals (Fig. 5a, b). A three-way ANOVA (Fig. 5a) for repeated measures with protein (EAAT2, Shank3, GluA2, and GABA_A_ α1) as a within-subject factor and infection status and treatment as between-subject factors revealed a highly significant interaction of infection status and treatment (*F*_1,15_ = 12.85; *p* = 0.003). Post hoc two-sample *t* tests showed all glutamatergic synapse components tested were similar to basal level (infected + untreated vs. infected + treated; EAAT2: *t*_3.8_ = − 4.55, *p* = 0.012; Shank3: *t*_6_ = − 3.54, *p* = 0.012; GluA2: *t*_6_ = − 4.41, *p* = 0.005), whereas an only trend-wise difference was observed for GABA_A_α1 (*t*_6_ = − 2.33, *p* = 0.059). Importantly, no differences between treated and untreated non-infected animals were observed (all *p* > 0.097).

#### Downregulation of *T*. *gondii*-related neuroinflammation after sulfadiazine treatment

##### Effects on inflammation-related astrogliosis

**Fig. 6:**
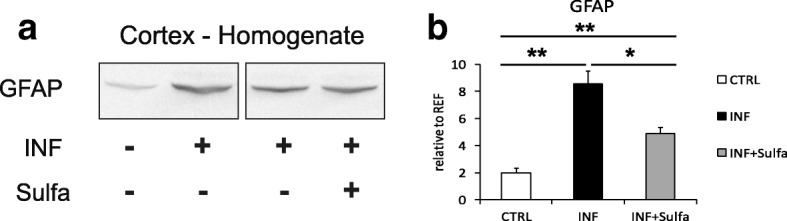
Reduced astrocyte activation upon sulfadiazine treatment. **a** Immunoblots of the cortical homogenates of uninfected control (INF−, CTRL), infected (INF+, INF), and infected animals treated with sulfadiazine (INF+, Sulfa+), and their according quantification (**b**). The upregulation of glutamatergic proteins (Fig. 5) after sulfadiazine treatment is complemented by downregulation of GFAP in the cortical homogenate, consistent with a reduction of global neuroinflammation in the brain upon drug treatment. Control mice (CTRL) *n* = 4, infected mice (INF) *n* = 4–5. Displayed results are mean values ± SEM. **p < 0.01, *p < 0.05

Immunoblot analysis in the cortex homogenates (Fig. 6a) revealed a reduction of GFAP in sulfadiazine-treated compared to untreated infected mice (Fig. 6b). A one-way ANOVA with a group (infected + untreated, *n* = 8; infected + treated, *n* = 4; control, *n* = 6) as a between-subject factor yielded a highly significant main effect of group (*F*_2,15_ = 21.54; *p* < 0.001). Post hoc two-sample *t* tests showed that GFAP abundance was significantly reduced in sulfadiazine-treated compared to untreated infected mice (infected + treated vs. infected + untreated: *t*_10_ = − 2.74, *p* = 0.021), albeit not to the level of uninfected mice (infected + treated vs. control: *t*_10_ = 5.04, *p* = 0.001).

##### Modulation of cytokine expression

**Fig. 7:**
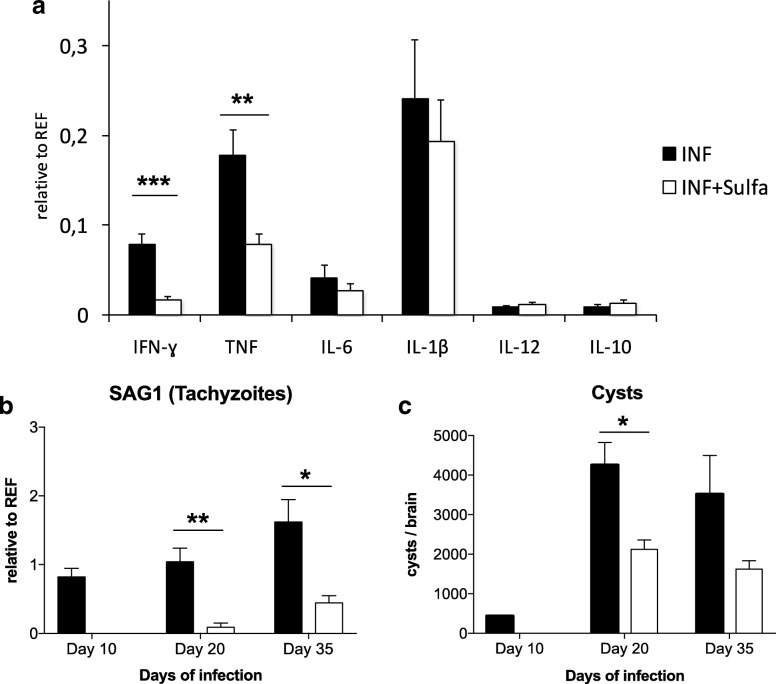
Sulfadiazine treatment is associated with reduced *Toxoplasma*-tachyzoite levels as well as abated neuroinflammatory response. **a** Quantitative real-time PCR (qRT-PCR) was performed on sulfadiazine-treated brain samples. Treatment-related changes in the expression of IFN-γ, IL-12, IL-10, TNF, IL-6, and IL-1β were compared to infected mice (INF) *n* = 9; infected, sulfadiazine-treated mice (INF + Sulfa) *n* = 9. **b** Quantitative real-time PCR (qRT-PCR) from indicated brain samples. Treatment related changes for SAG1 were also observed for day 10, day 20, and day 35. SAG1-tachyzoite surface antigen 1. Infected mice (INF) *n* = 4; infected, sulfadiazine-treated mice (INF + Sulfa) *n* = 4. **c** Time course for cyst enumeration in infected and sulfadiazine-treated brain samples after infection for 10, 20, or 35 days. Infected mice (INF) *n* = 3; infected, sulfadiazine-treated mice (INF + Sulfa) *n* = 4. Displayed results are mean values ± SEM. *p < 0.05, **p < 0.01, ***p < 0.001

In order to elucidate the expected downregulation of *T*. *gondii*-mediated neuroinflammation by sulfadiazine treatment (Fig. 7), we assessed the expression of six cytokines of interest (IFN-γ, IL-12, IL-10, TNF, IL-6, IL-1β, Fig. 7a) in the brain tissue using quantitative real-time PCR (qRT-PCR). A MANOVA with treatment (sulfadiazine vs. untreated) as a fixed factor, experiment as a covariate, and cytokine levels as dependent variables revealed a main effect of treatment (Wilks’ *λ* = 0.234; *F*_6,10_ = 5.44; *p* < 0.010). Tests of within-subject factors showed this effect to mainly reflect a difference in TNF and IFN-γ levels (all *p* < 0.002). Post hoc two-sample *t* tests to assess directionality revealed a significant downregulation of TNF (*t*_10.9_ = − 3.32, *p* = 0.008) and IFN-γ (*t*_9.7_ = − 4.95, *p* = 0.001) in sulfadiazine-treated mice.

##### Parasite cyst numbers and *T*. *gondii*-specific antigen expression

Finally, we aimed to verify the effects of sulfadiazine on fast-replicating tachyzoites and latent cysts. We first assessed the expression levels of the tachyzoite-specific surface antigen-1 (SAG1), using qRT-PCR ([58]; see Fig. 7b). The expression of SAG1 was downregulated in sulfadiazine-treated animals from day 20 onwards (main effect of treatment: *F*_1,28_ = 26.37; *p* < 0.001; two-way ANOVA with treatment and day as fixed factors). Post hoc two-sample *t* tests further confirmed that SAG1 expression was markedly reduced in treated compared to untreated animals on day 20 (*t*_4.7_ = 4.53; *p* = 0.007) and 35 (*t*_7.3_ = 3.43; *p* = 0.010).

*T*. *gondii* cyst count (Fig. 7c) was performed in sulfadiazine-treated and untreated infected brains on days 10, 20, and 35. Type C intraclass correlation coefficients (ICCs) for cyst count were uniformly high (0.826 < ICC < 0.987; all *p* < .039), confirming high inter-rater reliability [59]. Two-way ANOVAs with treatment and day as fixed factors and cyst count as dependent variable showed a main effect of treatment (all *F* > 12.08; all *p* < 0.005), whereas an effect of day was only observed when day 10 was included (*F*_2,15_ = 22.27, *p* < 0.001), reflecting an increase in cyst count from day 10 to day 20. Post hoc two-sample *t* tests showed a significant difference between untreated and treated animals on day 20 (*t*_5.3_ = 3.45; *p* = 0.017), but not on day 35 (*p* = 0.188).

## Discussion

We have obtained converging evidence for an interference of chronic *T*. *gondii* infection with the protein composition of synapses by combining proteomics and candidate-based protein expression studies in synaptosomes. Immunoblot and immunofluorescence data point to a marked interference of *T*. *gondii*-related neuroinflammation with synaptic protein composition. The recovery levels of glutamatergic synaptic protein abundances upon sulfadiazine treatment of infected mice, which were similar to basal levels, support the view that neuroinflammation affects synaptic protein composition and thereby, most likely, function. These effects were more pronounced for glutamatergic transmission in both the proteomic (Fig. 1, Additional file 3, Additional file 6) *and* candidate-based investigations (Figs. 3 and 4), but the proteomic data also indicate a reduction in GABAergic synapse components in synaptosomal preparations (Additional file 7). Our investigation was largely based on purified synaptosomal fractions, a well-established approach which has been “exhaustively characterized in functional terms” [60, 61]. Importantly with respect to our results, previous investigations of synaptosomal protein composition in the developing mouse brain have revealed that, besides pre- and postsynaptic proteins, synaptosomes also contain multiple inflammation-related proteins [62]. Among those proteins were interferon-inducible immunity-related GTPase Irgm1, interleukin enhancer-binding factor 2 (ILF2) as well as glial proteins like GFAP and proteoglycans of the synapse surrounding extracellular matrix proteoglycans. The presence of proteins indicating immune response has been also highlighted in a recent publication on proteomic analyses of the human frontal lobe tissue from *T*. *gondii*-infected humans co-infected with HIV [63]. The quality of our proteomic data on changes in the glutamatergic and GABAergic systems as well as calcium homeostasis and extracellular matrix components is strongly supported by the same study. There is an overlap of 98 proteins (approx. one third) found in our screening with the data reported there. Strikingly, these common proteins are almost exclusively synaptic proteins comprising of glutamate and GABA receptors, neurotransmitter and/or ion transporters, calcium channels, extracellular matrix components, and neuronal cell adhesion molecules. A recent proteomic study used the same synaptosomal approach in a mouse model for neurodegeneration [64]. In a meta-analysis, a set of more than 130 common biological processes between both datasets became obvious and might be indicative for certain similarity to our experimental system, underlining a multipartite concept of the synapse [65].

### Alterations of the synaptosomal proteome

Evidently, a large number of synaptosomal proteins was found upregulated in the proteomic investigations, and meta-analyses revealed that this was mostly observed for proteins involved in MHC class I-dependent antigen presentation, protein processing in the ER, and, most prominently, poly(A)-RNA binding. The latter observation is compatible with a recent transcriptome study demonstrating upregulation of proteins related to nuclear RNA processing in *T*. *gondii*-infected primary neural cultures [48]. Polyadenylation is a critical step in mRNA synthesis and serves to stabilize mRNAs after transcription. The most straightforward explanation for the increased abundance of proteins involved in poly(A)-RNA binding may be an overall increase in protein synthesis or turnover, which in turn may be related to increased expression of proteins related to inflammatory and immunological processes, such as MHC class I-dependent antigen recognition, which also showed a significant upregulation (Fig. 1). Poly(A)-RNA processing has been increasingly implicated in synaptic plasticity and neurodegenerative disorders [66, 67], and disruption of normal synaptic poly(A)-RNA processing leads to impaired synaptic protein synthesis and cognitive dysfunction [68]. Future research should thus assess the functional consequences of up regulation of poly(A) RNA-binding proteins in *T*. *gondii*-induced neuroinflammation. Interestingly, our proteomic data confirm to a large degree results from a previous study using full genome microarrays to analyze RNA levels in chronically *T*. *gondii*-infected mouse brains [69].

Although fewer proteins were downregulated rather than upregulated, a remarkable observation is the clustering of downregulated proteins in excitatory and inhibitory synaptic transmission, plasticity, and learning. This observation expands previously reported transcriptome alterations in pathways related to epilepsy and neurodegeneration and also malignancy [48]. In that study, pathways related to synaptic transmission and plasticity were not reported among the most strongly affected functional systems. One reason for this may be that synaptic changes at the mRNA level may be more transient and thereby less readily detectable than the more long-lasting changes in protein composition. Additionally, differential alterations of protein turnover rates caused, e.g., by changes in protein stability or degradation may contribute to variations in protein composition, while not being detectable at the mRNA level. Furthermore, Ngô and colleagues investigated primary neuronal cultures, while the present study was conducted using synaptosomes prepared from in vivo infected mice and may therefore be more likely to capture the effects of *T*. *gondii* infection on the molecular organization of synapses in otherwise normally developing brains, i.e., under more physiological—or pathophysiological—conditions.

The results of meta-analyses using IPA™ are summarized in Additional file 9. In addition to confirming functional aspects extracted from the proteomic datasets already revealed by DAVID and GeneCodis analyses, additional strong links to neurological diseases and psychiatric disorders emerge. This is also indicative of a functional relevance of our data as they are in agreement with earlier observations in which *T*. *gondii* infection status correlates with higher incidences of neurological and psychiatric disorders [8, 17, 70]. Concerning molecular and cellular functions, we found our proteomics data strongly related to changes in cell-to-cell signaling and interaction, cell morphology, cellular development, and also cellular assembly and organization. At the physiological level, the dataset identifies major aspects of nervous system development and function as changed by persistent *T*. *gondii* infection (Additional file 9). Further IPA™ results point to increases in the antigen presentation pathway (Additional file 10) and the lipid antigen presentation by the CD1 pathway (Additional file 11). Our proteomics data also revealed a significant reduction of synaptic proteins related to glutamatergic transmission (Additional file 6) and to GABAergic signaling (Additional file 7).

### Interference of *T*. *gondii* with glutamatergic neurotransmission

Downregulation of synaptosomal proteins in *T*. *gondii*-infected mice was most pronounced for proteins involved in calcium signaling, glutamatergic and GABAergic synapse function, and, generally, in neural plasticity and learning and memory. Regarding glutamatergic transmission, major results of our proteomic investigations were confirmed by candidate-based experiments. In the synaptosomes, we observed downregulation of EAAT2, Shank3, and the AMPA-type glutamate receptor subunit GluA2. In the hippocampal and neocortical tissue homogenates, we found reduced abundances of the EAAT2, Shank3, the AMPA receptor subunit GluA1, and the NMDA receptor subunit GluN1, respectively. Infection-related downregulation of EAAT2 has been observed previously in the forebrain homogenates of mice with chronic toxoplasmosis [45]. Beyond confirming those results, we have shown that this downregulation was particularly pronounced in synaptosomes. Furthermore, using immunofluorescence staining, we have also demonstrated infection-related alterations of EAAT2 distribution throughout the brain. The downregulation of major glutamatergic synapse components (GluA2 and Shank3) observed here calls into question whether chronic *T*. *gondii* infection merely results in excess glutamatergic signaling due to reduced uptake as suggested [45, 71]. Instead, our results point to a more global impairment of glutamatergic synapse function related to *T*. *gondii*-induced neuroinflammation. One explanation for this is the simplification of neuronal architecture and a significant reduction of spine density [8] and spine numbers in the prefrontal cortex [45]. Another possibility could be a negative feedback effect, that is, excess synaptic glutamate might result in adaptive downregulation of proteins involved in neurotransmitter release as well as glutamate receptors and their interacting proteins. In line with this interpretation, both presynaptic scaffolding proteins like Piccolo, Bassoon, or RIM and postsynaptic adapter molecules like PSD-95/Dlg4, SAPAPs/DLGPs, or Shank proteins were found among the top 100 downregulated proteins in our proteomic investigations. Furthermore, changes detected in synaptic proteomes related to GABAergic transmission might also contribute to chronic *T*. *gondii* infection-associated alterations in behavior or correlations to neuropsychiatric disorders as a consequence of subtle alterations in the balance of excitatory and inhibitory neurotransmission.

With respect to the reduced expression and changed distribution of EAAT2 in infected mice, it remains yet to be determined, to what extent that reduction could be attributed to glial versus neuronal EAAT2. While the molecule is most strongly expressed in glial cells, it also shows a certain level of neuronal expression [72]. Neuronal EAAT2 is actually mainly found at axon terminals, particularly as part of synaptosomal preparations [73] and may therefore have contributed substantially to the reduced EAAT2 expression observed in the synaptosomes of infected mice. On the other hand, in the present study, EAAT2 was also reduced in the brain homogenates. Considering that, at least under standard conditions, 80% of EAAT2 is expressed in astrocytes [73], it is thus implausible to assume that the reduced EAAT2 abundances in infected animals purely reflected a reduction of neuronal EAAT2. In this context, it should also be noted that at least a fraction of the EAAT2 protein found to be reduced in synaptosomes may actually be of glial origin, as astrocytes can form protrusions into the synaptic cleft, thereby allowing rapid EAAT2-dependent synaptic glutamate clearance [74]. These astrocytic “endfeet” are known to copurify partly with synaptosomes, suggesting that also in the synaptosomal preparations, both neuronal and glial EAAT2 abundances were most likely reduced upon *T*. *gondii* infection. It should be noted that downregulation of EAAT2 was paralleled by upregulation of the glial fibrillary acidic protein (GFAP), which is exclusively expressed in astrocytes (Fig. 6). This observation is noteworthy as it indicates that, at least with respect to astrocytes, the observed alterations in synaptosomal protein composition could not be attributed to a mere change in predominant cell types.

While immunoblot analyses essentially confirmed this overall pattern of synaptic protein downregulation in *T*. *gondii*-infected mice, this could not be confirmed for all candidate proteins in the brain homogenates, which in turn might be indicative for a specific synaptic regulation of protein abundance. Particularly, GluN2B downregulation was not significant in the hippocampus and only moderate in the cortex when compared to other synaptic proteins. One explanation for this may be the relatively small number of animals investigated and the resulting lack of statistical power to detect subtle differences. Alternatively, it may be speculated that the GluN2B subunit is primarily a component of extrasynaptic NMDA receptors, whereas GluN1 is a prominent component of synaptic NMDA receptors [75]. A robust downregulation of synaptic NMDA receptors, accompanied by a less pronounced downregulation of extrasynaptic NMDA receptors, might shift the balance between synaptic and extrasynaptic NMDA receptor-dependent glutamate signaling—a molecular mechanism that has been implicated in learning impairment or ultimately excitotoxicity [76].

Although we could confirm and expand previous observations regarding dysregulation of glutamatergic neurotransmission in chronic toxoplasmosis [8, 45, 71], results of the GABAergic signaling are less clear. In analogy to the changes observed in the glutamatergic system, reductions of GABAergic synapse components in infected mice were observed in our proteomic analyses. However, in immunoblot analysis, synaptic expression levels of GABA_A_-α1 receptor subunits were not significantly different between infected and control animals. Considering the superior sensitivity of MS-based techniques over the traditional immunodetection, there is little reason to question the changes observed in the GABAergic system. In a previous study by Brooks et al. [47], no changes in global GAD67 expression levels had been found, but loss of the typical synaptic localization of GAD67 was observed. We therefore suggest that GABAergic alterations in *T*. *gondii*-infected mice may be rather subtle, but its impact should not be underestimated, particularly in conjunction with the more pronounced alterations in glutamatergic neurotransmission. Hence, changes in the balance between excitation and inhibition in the chronic infection status are very likely and might contribute to pathophysiological changes implicated in neuropsychiatric disorders [71].

### Inflammation-related modulation of synaptic and neural protein expression patterns

In contrast to the downregulation of proteins related to synaptic transmission in synaptosomes of *T*. *gondii*-infected mice, inflammation-related proteins were strongly upregulated after infection (Fig. 1). MS results revealed an infection-related increase of the synaptosomal levels of proteins like interferon-inducible GTPase 1 (IIGP1), immunity-related GTPase (IGTP), interferon-induced protein with tetratricopeptide repeats 3 (IFIT3), and guanylate-binding protein 2 (GBP2), which are all signature molecules for the infection with the intracellular pathogen *T*. *gondii*. Moreover, the signaling molecule signal transducer and activator of transcription 1-alpha/beta (STAT1) was elevated during chronic infection, indicating an active host defense mechanism. The astrocyte marker glial fibrillary acidic protein (GFAP) was also strongly upregulated, most likely indicating inflammation-related astrocyte activation. This finding was corroborated by our immunoblot analyses demonstrating GFAP upregulation in tissue homogenates. The rather uniform increase of GFAP levels is indicative of a general response to the infection. GFAP is exclusively expressed in astrocytes, and its upregulation has been demonstrated during brain development, regeneration, or reactive gliosis [57]. Increased GFAP expression has also been suggested as an indicator of neuroinflammatory responses [56] and that might also apply to our current observations (Fig. 6) [57]. Thus, locally restricted parasite cysts in a small number of infected neurons may trigger a systemic inflammatory response.

Pro-inflammatory cytokines are critically involved in the physiology and pathophysiology of neuroinflammatory responses. Our qRT-PCR results are compatible with the previously reported pivotal role of IFN-γ and TNF in the neuroinflammatory response to *T*. *gondii*. IFN-γ, which is the key cytokine to control *T*. *gondii* infection, is produced at high levels initially by microglia and neutrophil granulocytes and by lymphocytes at later stages of the infection [6, 77]. Accumulating evidence suggests a role for IFN-γ in synaptic plasticity and neurodegeneration [38]. Upregulation of IFN-γ levels upon infection, as observed in our study, might effect MHC-1 expression by neurons and subsequent synaptic pruning by innate immune cells [78, 79]. Elimination of the synapses by mononuclear immune cells follows similar patterns and has been reported in diseases like multiple sclerosis [80] and Alzheimer’s [81]. More evidence suggests interference of IFN-γ with glutamatergic signaling, with AMPA receptors being a key player in IFN-γ-triggered excitotoxicity and neurodegeneration. Complex formation of active IFN-γR and GluA1 can result in calcium influx and production of nitric oxide, followed by dendritic bead formation [82]. Especially, the effect of chronic IFN-γ was shown to modulate AMPA receptor expression in the hippocampal neurons, which would be a possible explanation for our results, as sulfadiazine treatment was associated with reduced IFN-γ levels and synaptic protein expression in infected mice, similar to basal levels. In addition to IFN-γ, the specific inflammatory milieu with TNF, IL-6, and IL-1β might be responsible for the observed neuronal changes. Previous reports, reviewed by Klein et al. [33], indicate the involvement of IFN-γ and IL-1β in adult hippocampal neurogenesis as well as in learning and memory. Alterations include inhibitory (IL-1β, IFN-γ) or promoting (TNF) effects on long-term potentiation (LTP) as well as promoting (IFN-γ, TNFR2) or inhibiting (TNFR1) effects on neurogenesis in adults. Sulfadiazine treatment resulted in reduced tachyzoite numbers which was followed by downregulation of the cytokines IFN-γ, TNF, and IL-6, and IL-1β levels were sligthly affected. Thus, it is likely that the changes in the inflammatory milieu could influence neuronal alterations and synaptic protein expression.

### Sulfadiazine treatment results in decreased SAG1 levels

The antiparasitic drug sulfadiazine is a para-aminobenzoic acid inhibitor, interfering with the folic acid synthesis pathway of the fast-replicating tachyzoites. Our results indicate that the sulfadiazine treatment, starting on day 10 post-infection, diminished tachyzoite (SAG1) levels and reduced parasite dissemination to the CNS. Cyst numbers were also affected, as cyst development starts as early as the tachyzoites enter the CNS, infecting neurons, and continues alongside the infection. Thus, reduced tachyzoite levels after treatment resulted in partially diminished cyst development as reported previously [69]. Cyst levels reached high numbers on day 20 and remained comparable on day 35 as previously indicated in studies with C57BL/6 mice [83, 84]. Importantly, cyst numbers on day 35 were not significantly reduced by the sulfadiazine treatment, only a trend could be observed, indicating the comparable principal presence of intraneuronal cysts in both experimental groups.

### Synaptic protein levels after sulfadiazine treatment of infected mice

Protein levels of EAAT2, Shank3, and GluA2 were at least partly restored by treatment of infected animals with the sulfonamide antibiotic sulfadiazine, and a similar trend could also be observed for GABA_A_ receptor α1 subunits. GFAP levels, on the other hand, were lower in sulfadiazine-treated compared to untreated animals, compatible with a reduction of the infection-related neuroinflammatory response. Since sulfadiazine reduces tachyzoites and infection-related neuroinflammation, but not cysts, we suggest that the *T*. *gondii*-induced changes in synaptic protein composition are most likely an indirect effect mediated by inflammatory agents. A previous study associated the larger volume of inflammatory infiltrates, observed by histopathology with more pronounced abnormal behavior in mice chronically infected with *Toxoplasma gondii* [69]. This study described for the first time in details the neuroinflammatory changes in outbred mice upon infection. IFN-γ and TNF are both critically involved in the control of *T*. *gondii* infection [85] and were downregulated by sulfadiazine treatment in our present study. They have previously been shown to impair synaptic plasticity mechanisms like LTP [32, 86]. Therefore, our results suggest that neuroinflammation-related impairment in neural plasticity may partially result from subtle modulations of synaptic ultrastructure. It is a candidate pathophysiological mechanism that may also be relevant for neuroinflammation of another origin than *T*. *gondii* infection.

### Clinical implications

With the advance of genome-wide association studies (GWAS), several components of the glutamatergic synapse have been identified as risk factors for the major psychiatric disorders like schizophrenia, bipolar disorder, and major depression. These include neurocan [52, 87], Homer-1 [53], and Piccolo [88], all of which were downregulated in synaptosomes of *T*. *gondii*-infected animals. Similarly, the genes of the Shank proteins, which also showed reduced abundance in infected animals, have been linked to autism spectrum disorders [89, 90]. Moreover, proteins related to MHC class I-dependent immune responses were upregulated in infected animals, and GWAS for schizophrenia have actually detected a cluster of risk loci in genes related to the MHC class I complex [91]. Similar associations have been found for bipolar disorder [92]. This dual overlap of genetic findings in the major psychoses with alterations of synaptic protein composition in latent toxoplasmosis—which is considered a risk factor for the very same disorders—convergingly raises the possibility that inflammation-induced changes in synaptic ultrastructure and ultimately function may constitute an overarching pathomechanism, by which diverse genetic and environmental factors may affect disease risk.

## Conclusions

Our results suggest that chronic *T*. *gondii* infection induces distinct alterations in synaptic protein composition, with downregulation of a large number of proteins involved in synaptic plasticity, learning, and risk for neuropsychiatric disorders. These synaptic alterations are accompanied by an inflammatory response mediated by upregulation of several pro-inflammatory marker proteins. Elimination of tachyzoites results in reduction of inflammatory response in reversal of synaptic changes, suggesting that the *T*. *gondii*-induced specific inflammatory milieu should be considered the direct causal factor for neuronal alterations.

## Additional files

Additional file 1: Supplementary methods [50, 88, 90, 93]. (DOCX 31 kb)

Additional file 2: Overview of selected brain areas for quantification. The depicted brain only serves as a showcase for the quantification approach of each brain. Selected regions for the cortical and subcortical areas were cortices (Cx1 and Cx2), striata (Str1 and Str2), and thalamus (Th) from both hemispheres. The hypothalamic region (HTH) between anterior commissures was selected as the reference area for calculations according to the scheme. Areas for quantification were selected for both, control and infected animals, accordingly. However, areas of saturated intensity were identified as artifacts and excluded from quantification. Integrated density was used as a measurement of intensities. For each area, intensities relative to the reference area were calculated, and in case of cortex and striatum, added together. (TIF 2160 kb)

Additional file 3: Proteomic analysis of synaptosomes from *T*. *gondii* infected mice. Compilation of 292 significantly regulated proteins found at minimum in three biological replicates. (XLS 1760 kb)

Additional file 4: Proteomic analysis of synaptosomes from *Toxoplasma gondii*-infected mice. Heatmaps showing relative protein abundances (log_2_) of all 292 significantly regulated proteins in synaptosomes after *Toxoplasma gondii* infection in four separate sample pairs. Color codes are indicated. (TIF 384 kb)

Additional file 5: Proteomic analyses revealed robust quantification of protein abundances in isolated synaptosomes. Venn diagram showing all proteins found in four separate sample pairs (1–4). Numbers (dark gray) indicate the number of proteins found in corresponding sample pairs. Total numbers of found proteins in each single sample pair are given in parentheses. (PDF 2370 kb)

Additional file 6: Glutamate receptor signaling pathway according to IPA™. Symbols are explained in a table (part B). Filled symbols represent proteins found to be altered in synaptosomes according to our MS data, green indicates reduced levels, and red notifies increased levels compared to controls. (PDF 1650 kb)

Additional file 7: GABA receptor signaling pathway according to IPA™. Symbols are explained in a table (part B). Filled symbols represent proteins found to be altered in synaptosomes according to our MS data, green indicates reduced levels, and red notifies increased levels compared to controls. (PDF 1430 kb)

Additional file 8: GABA_A_α1 expression upon chronic *Toxoplasma* infection. A) Immunoblot analysis from cortical synaptosomes revealed a downward trend after infection for GABA_A_α1. B) Immunoblot analysis from brain homogenates in the neocortex and hippocampus for GABA_A_α1 shows no significant changes. C) Coronal sections of *Toxoplasma gondii*-infected (INF) and control animals (CTRL), stained with markers for GABA_A_α1, with focus on specific cortical and subcortical areas (neocortex, thalamus, striatum). Integrated density from each quantified area was normalized to hypothalamic reference area (see Additional file 2) and showed no significant changes in staining for GABA_A_α1. Control mice (CTRL) *n* = 3, infected mice (INF) *n* = 3. Scale bar 1 mm. Displayed results are mean values ± SEM. (TIF 7200 kb)

Additional file 9: Affected top canonical pathways, diseases and disorders, molecular and cellular functions, and physiological system development and function after chronic *T*. *gondii* infection according to Ingenuity Pathway Analysis (IPA™). Ingenuity Pathway Analysis (IPA™) of our data set according to Additional file 3 reveals a large number of canonical pathways, diseases and disorders, molecular and cellular functions, and physiological system development and function represented in our data with high significance. These findings agree with former observations related to *T*. *gondii* infection. (TIF 3320 kb)

Additional file 10: Antigen presentation pathway according to IPA™. Symbols are explained in a table (part B). Filled symbols represent proteins found to be altered in synaptosomes according to MS data, green indicates reduced levels, and red notifies increased levels compared to controls. (PDF 1690 kb)

Additional file 11: Lipid antigen presentation pathway according to IPA™. Symbols are explained in a table (part B). Filled symbols represent proteins found to be altered in synaptosomes according to MS data, green indicates reduced levels, and red notifies increased levels compared to controls. (PDF 1830 kb)

## Abbreviations

ACN: Acetonitrile
AMPA: α-amino-3-hydroxy-5-methyl-4-isoxazolepropionic acid
ANOVA: Analysis of variance
BCA: Bicinchoninic acid
CD1: Cluster of differentiation 1
CNS: Central nervous system
CTRL: Controls
EAAT2: Excitatory amino acid transporter 2
ECM: Extracellular matrix
FDR: False discovery rate
GABA: γ-aminobutyric acid
GAD67: Glutamate decarboxylate 67
GBP2: Guanylate-binding protein 2
GFAP: Glial fibrillary acidic protein
GLT-1: Glutamate transporter 1
GluA1: Glutamate receptor subunit 1
GluA2: Glutamate receptor subunit 2
GluN1: Glutamate ionotropic receptor NMDA type subunit 1
GluN2B: Glutamate ionotropic receptor NMDA type subunit 2B
GWAS: Genome-wide association studies
HIV: Human immunodeficiency virus
HRP: Horse radish peroxidase
HS: Horse Serum
HTH: Hypothalamus
ICC: Type C intraclass correlation coefficients
ID: Integrated density
IFIT3: Interferon-induced protein with tetratricopeptide repeats 3
IFN-γ: Interferon gamma
IIGP1: Interferon-inducible GTPase 1
IL-12, IL-1, IL-6, IL-1ßγ: Interleukin-12, interleukin-1, interleukin-6, interleukin-1ßγ
ILF2: Interleukin enhancer-binding factor 2
INF: Infected
Irgm1: Interferon-inducible immunity-related GTPase
iTRAQ: Isobaric tags for relative and absolute quantitation
KEGG: Kyoto Encyclopedia of Genes and Genomes
LC-MS/MS: Liquid chromatography-mass spectrometry/mass spectrometry
MAP 2: Microtubule-associated protein
MHC: Major histocompatibility complex
NMDA: *N*-methyl-d-aspartate
NMRI: Naval Medical Research Institute
NO: Nitric oxide
PBS: Phosphate-buffered saline
PSD-95/Dlg4: Postsynaptic density protein 95
qRT-PCR: Quantitative real-time PCR
Ref: Reference area
RIM: Rab3-interacting molecule
ROI: Region of interest
SAG1: Surface antigen 1
SAPAPs/DLGPs: Disks large-associated proteins
SDS-PAGE: Sodium dodecyl sulfate-polyacrylamide gel electrophoresis
Shank3: SH3 and multiple ankyrin repeat domains 3
STAT1: Signal transducer and activator of transcription 1
Sulfa: Sulfadiazin
*T*. *gondii*: *Toxoplasma gondii*
TBS-A: Tris-buffered saline + sodium azide
TBS-T: Tris-buffered saline + Tween 20
TEAB: Triethylammonium bicarbonate
TFA: Trifluoroacetic acid
TGFß-1: Transforming growth factor beta-1
TNF: Tumor necrosis factor
UPLC: Ultra performance liquid chromatography
