# Supplementary material for: Chronic Toxoplasma infection is associated with distinct alterations in the synaptic protein composition
Source: J Neuroinflammation. 2018 Aug 1;15:216. doi: 10.1186/s12974-018-1242-1 (PMC6090988; doi:10.1186/s12974-018-1242-1)
Supplement: Supplementary file 1 — Supplementary methods [50, 88, 90, 93]. (DOCX 31 kb) [file 12974_2018_1242_MOESM1_ESM.docx]

Supplementary Methods

**Preparation of Synaptosomes**

Subcellular fractionation was performed as described previously [49], with minor modifications. In brief, brain tissue from the regions of interest was homogenized in Buffer A (5 mM HEPES/320 mM sucrose pH 7.4 containing cOmplete™ protease inhibitor cocktail (Roche, Indianapolis, IN)) at a concentration of 1g/10 ml, using a *K-Control TLC* homogenizer (Kaltenbach and Voigt, Biberach, Germany) with 12 strokes at 1000 rpm. All centrifugation steps were performed at 4°C. Homogenates were centrifuged twice at 1000 x g for 10 min in order to remove nuclei and cell debris. The combined supernatants S1 and S1’ from both centrifugation steps were centrifuged twice at 12000 x g for 20 min. Again, pellet P2 was re-homogenized in Buffer A (10 ml/g tissue) between the centrifugation steps. Finally, pellet P2’ was re-homogenized in 5mM Tris-HCl/320 mM sucrose pH 8.1 (1.5 ml/g tissue). This sample was loaded onto a sucrose step gradient (0.85 M/ 1.0 M/ 1.2 M) in 5mM Tris-HCl pH 8.1 and centrifuged at 85000 x g for 2 h. Synaptosomes were collected at the 1.0 M/1.2 M sucrose interface and stored at -80°C until further use.

**Proteomics: iTRAQ labeling and SCX chromatography**

iTRAQ labeling was performed according to the manufacturer´s protocol (Applied Biosystems). Briefly, 150 µg dried peptides were redissolved in 20 µl iTRAQ dissolution buffer at a ratio of less than 50 µg peptide/iTRAQ vial and incubated for 2 h, followed by vacuum drying. A small aliquot was used to confirm the minimum percentage of peptide labeling using a 30 min RP18 gradient and the OrbitrapVelos mass spectrometer. Labeled fractions with reporters 114(115) for infected and 116(117) for non-infected mice were combined and employed for SCX chromatography to reduce the complexity.

For subfractionation of labeled peptide mixtures were subfractionated as described previously [93] up to 100 µg of peptide were separated on a Mono S PC 1.6/5 column (GE Healthcare) via an Ettan micro-LC system (GE Healthcare) using a 30 min gradient from 0 to 35 % SCX buffer B (0.065 % formic acid (FA), 25 % ACN supplemented with 0.5 M KCl) at a flow rate of 150 µl/min. Up to twenty 150 µl fractions were collected, desalted, and used for LC-MS/MS.

**Statistical analyses**

***Statistical analyses of proteomics data***

In order to identify proteins that showed significantly different expression levels in relation to a variable of interest (here: *T. gondii* infection), we first computed the binary logarithm of the ratio between the protein amounts detected in treated versus untreated animals for each protein identified (log_2_ [prot_inf_ / prot_con_]; hence termed *regulation factor*). We then tested our MS results against the null hypothesis that the measured regulation factors occurred by chance and were thus randomly distributed. Owing to our low number of replicates (N ≤ 4), we applied a conservative model-based statistical approach based on the assumption that regulation factors are normally distributed and differ in their expected (mean) values, but have equal standard deviations (for a detailed description, see [89]). When the proteins are ordered as a function of their regulation factors in each replicate, we would expect random orders under the null hypothesis. Our test was applied to each protein, and for any given protein, each regulation factor represented an empirical quantile in the corresponding replicate. The test statistic was based on these quantiles. A *p* value of <1 was only computed when all regulation factors lay either above or below the median of the corresponding replicate. In that case, the quantile closest to the median was considered the test statistic. The raw (uncorrected) *p* value for the considered protein was twice the probability of obtaining values beyond this quantile under the null hypothesis in all measured replicates [*Note:* The factor 2 reflects the use of a two-tailed test statistic, in order to capture both up- and down-regulation of proteins].

In an alternative approach, we considered only those proteins that were measured in at least three replicates. To this end, we modified the above-mentioned test statistic whenever the protein was measured in all four replicates. In this case, we did not consider the quantile closest to the median but the second closest one, and the resulting raw (uncorrected) *p* value was the probability that at least three out for quantiles lay beyond the quantile second closest to the median. The latter approach takes into account that there might be one outlier among the four replicates, in which the protein is less significantly regulated. In all cases, the raw *p* values were corrected for family-wise error rate using the Holm-Bonferroni method [93].

***Statistical analysis of candidate-based investigations***

Immunoblot (IB) and immunofluorescence (IF) signals were normalized to appropriate control signals that were expected to be unaffected by *T. gondii* infection and/or neuroinflammation. IB signals were normalized to the tubulin signal obtained from the same animals, and IF signals were normalized to a region with predominantly unspecific background fluorescence in the respective slices. Statistical analysis of the normalized signals was performed in two steps. First, proteins were grouped by their functional systems whenever more than one protein belonging to a functional system (i.e. glutamatergic or GABAergic system) were investigated. ANOVAs for repeated measures were computed with protein as within-subject factor and infection and/or sulfadiazine treatment as between-subjects factors. The Greenhouse-Geisser correction for non-sphericity was applied whenever a within-subjects factor had more than two levels. Next, *post hoc* two-sample *t*-tests were computed to further elucidate the directionality of significant main effects and interactions. Owing to the relatively small sample sizes, unequal variances were assumed in all comparisons.

To assess the regulation of cytokine expression profiles by sulfadiazine treatment of *T. gondii*-infected mice, raw mRNA levels of these cytokines were obtained from two experiments (experiment 1: 4 treated, 4 untreated; experiment 2: 5 treated, 5 untreated) and normalized to HPRT mRNA. Normalized mRNA levels were submitted to a MANOVA with treatment as fixed factor and experiment as covariate (coded as 0 or 1) [*Note:* We used a MANOVA instead of an ANOVA for repeated measures when comparing the cytokine levels, as we could not assume a positive correlation of the dependent variables, i.e. the cytokines, here]. Because variance homogeneity could not be assumed due to the relatively small sample size, we adjusted the significance level for all *post hoc* tests to 0.025, two-tailed, as previously suggested for this case [87]. *Post hoc* two-sample *t*-tests were employed to assess directionality of regulation for the cytokines significantly regulated by sulfadiazine treatment.

Expression levels of *T. gondii* tachyzoite antigen SAG1 in mice with and without sulfadiazine treatment on Day 10, 20, and 35 were analyzed using a two-factorial univariate ANOVA with day and treatment group as fixed factors and SAG1 expression levels as dependent variable, followed by *post hoc* two-sample *t*-tests.

Statistics on *T. gondii* cyst count in sulfadiazine-treated and untreated animals were based on the mean count obtained from three to five independent raters. Type C intraclass correlation coefficients were computed to assess interrater reliability. After determining sufficient reliability, mean cyst counts were submitted to a two-factorial univariate ANOVA with day and treatment group as fixed factors and cyst count as dependent variable, followed by *post hoc* two-sample *t*-tests.
