# Supplementary figures and images for: Chronic Toxoplasma infection is associated with distinct alterations in the synaptic protein composition
Source: J Neuroinflammation. 2018 Aug 1;15:216. doi: 10.1186/s12974-018-1242-1 (PMC6090988; doi:10.1186/s12974-018-1242-1)

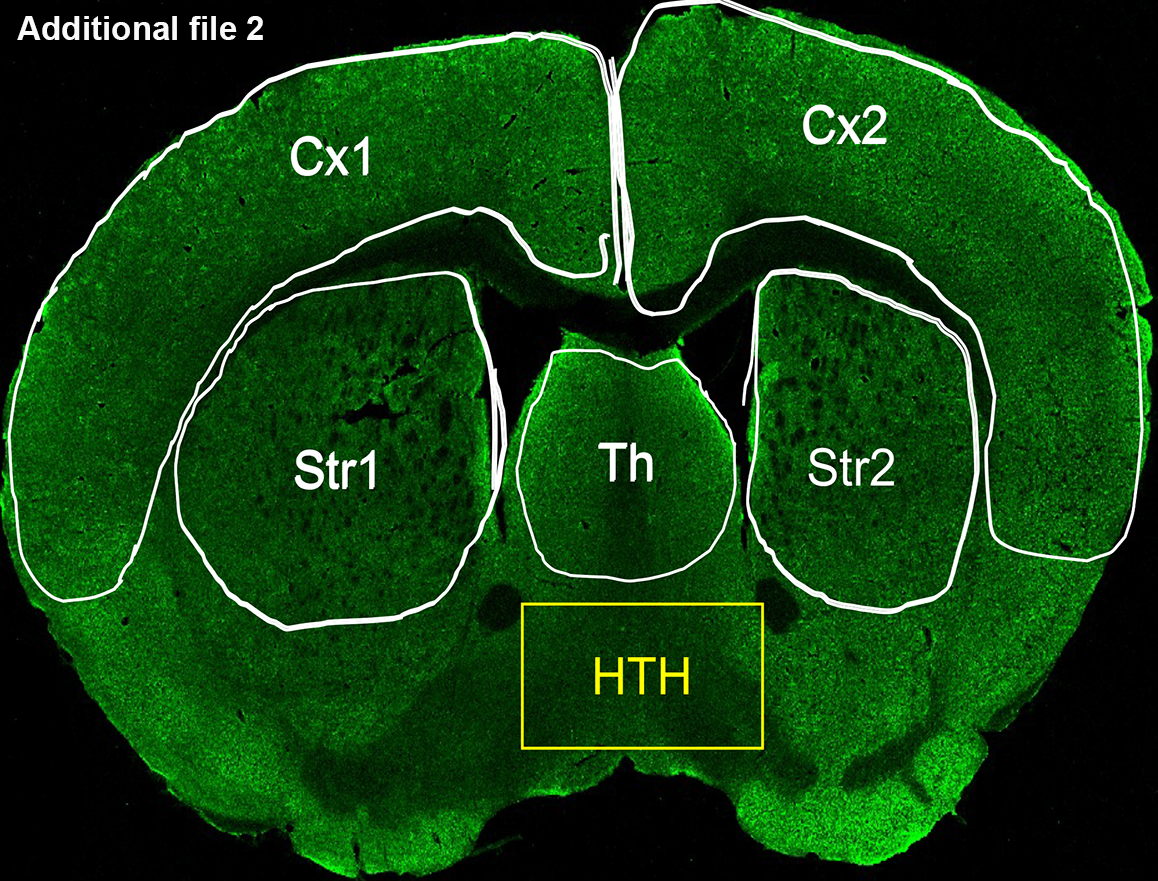

Supplement: Supplementary file 2 — Overview of selected brain areas for quantification. The depicted brain only serves as a showcase for the quantification approach of each brain. Selected regions for the cortical and subcortical areas were cortices (Cx1 and Cx2), striata (Str1 and Str2), and thalamus (Th) from both hemispheres. The hypothalamic region (HTH) between anterior commissures was selected as the reference area for calculations according to the scheme. Areas for quantification were selected for both, control and infected animals, accordingly. However, areas of saturated intensity were identified as artifacts and excluded from quantification. Integrated density was used as a measurement of intensities. For each area, intensities relative to the reference area were calculated, and in case of cortex and striatum, added together. (TIF 2160 kb) [file 12974_2018_1242_MOESM2_ESM.tif]

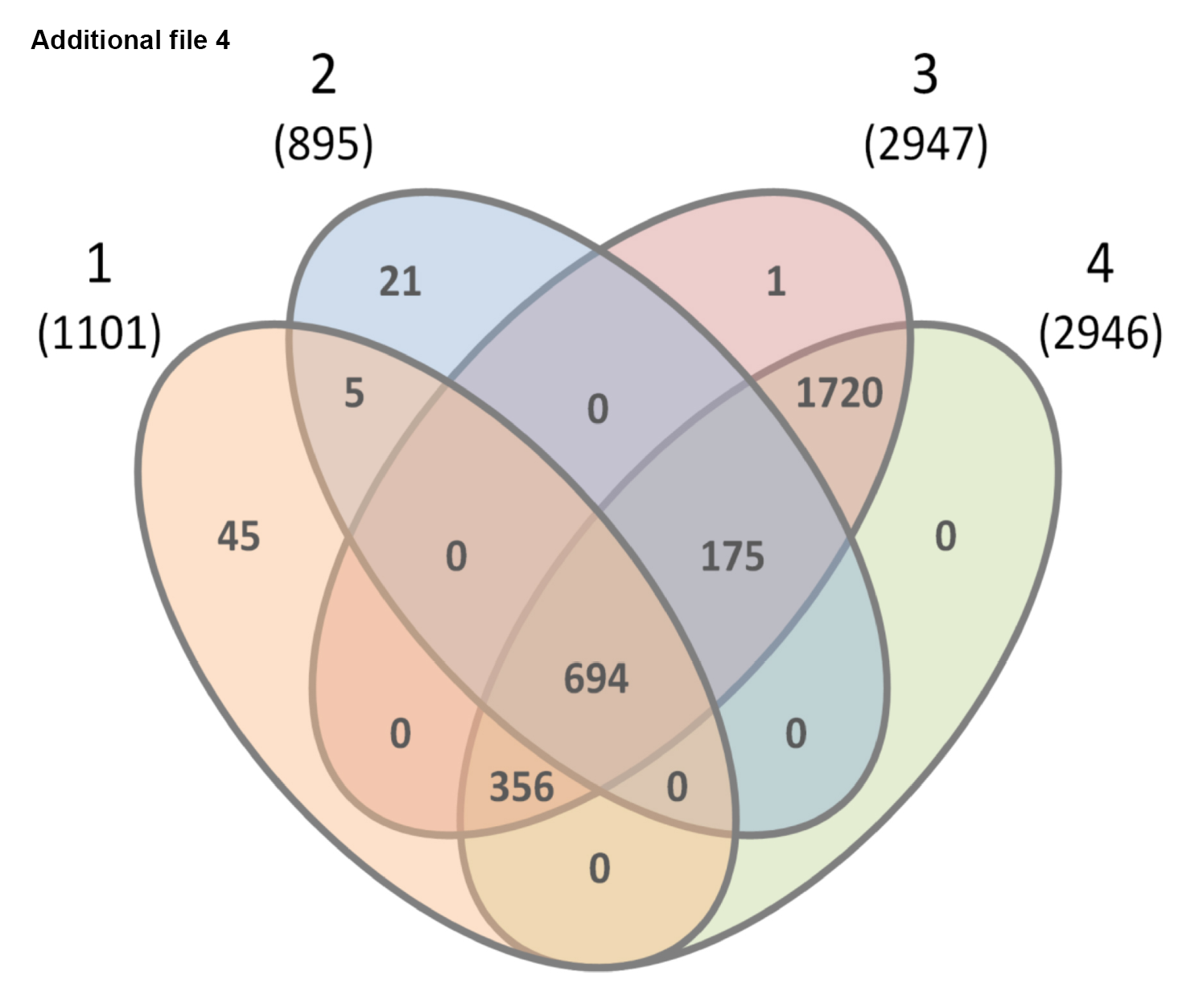

Supplement: Supplementary file 4 — Proteomic analysis of synaptosomes from Toxoplasma gondii-infected mice. Heatmaps showing relative protein abundances (log2) of all 292 significantly regulated proteins in synaptosomes after Toxoplasma gondii infection in four separate sample pairs. Color codes are indicated. (TIF 384 kb) [file 12974_2018_1242_MOESM4_ESM.tif]

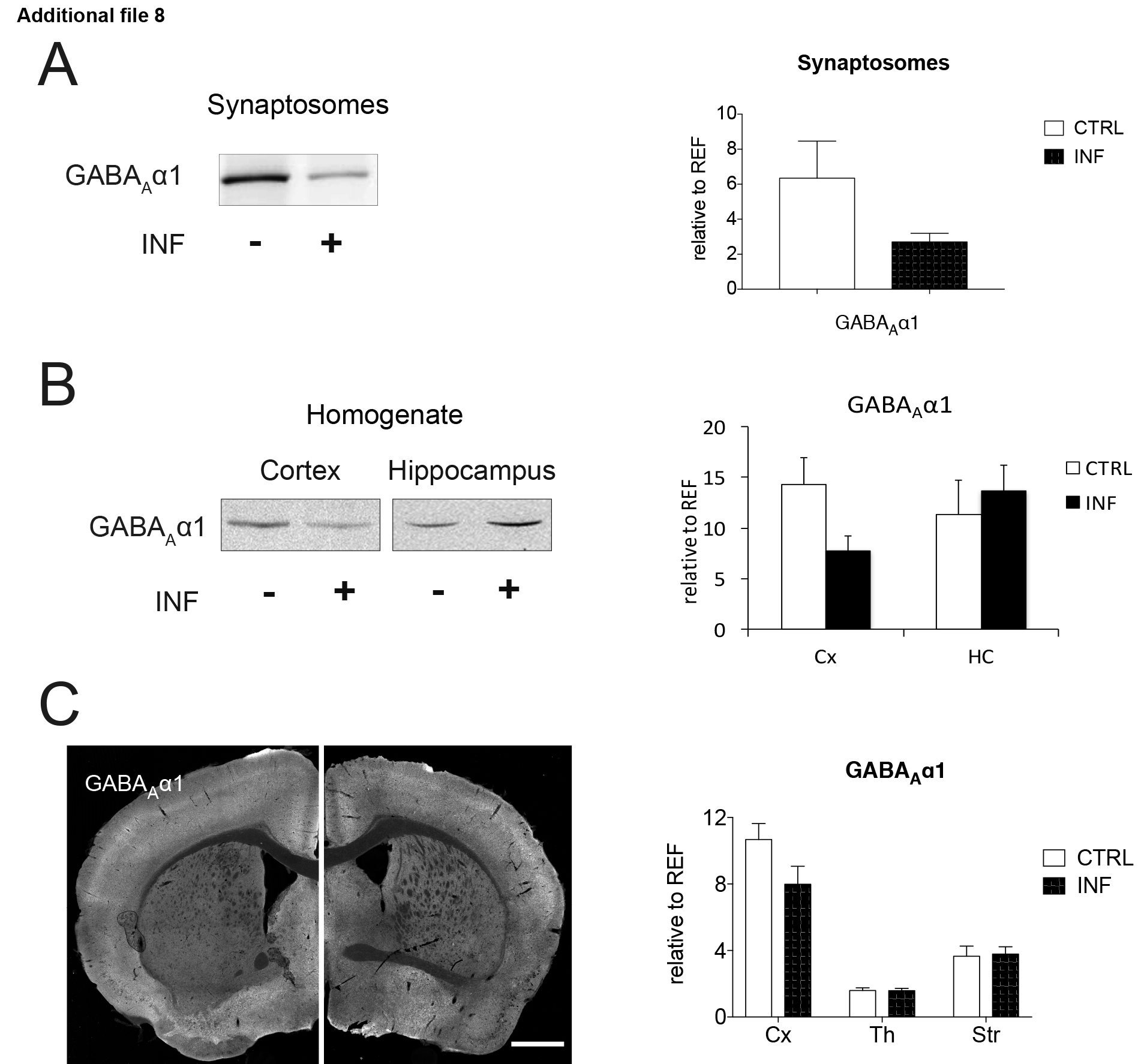

Supplement: Supplementary file 8 — GABAAα1 expression upon chronic Toxoplasma infection. A) Immunoblot analysis from cortical synaptosomes revealed a downward trend after infection for GABAAα1. B) Immunoblot analysis from brain homogenates in the neocortex and hippocampus for GABAAα1 shows no significant changes. C) Coronal sections of Toxoplasma gondii-infected (INF) and control animals (CTRL), stained with markers for GABAAα1, with focus on specific cortical and subcortical areas (neocortex, thalamus, striatum). Integrated density from each quantified area was normalized to hypothalamic reference area (see Additional file 2) and showed no significant changes in staining for GABAAα1. Control mice (CTRL) n = 3, infected mice (INF) n = 3. Scale bar 1 mm. Displayed results are mean values ± SEM. (TIF 7200 kb) [file 12974_2018_1242_MOESM8_ESM.tif]

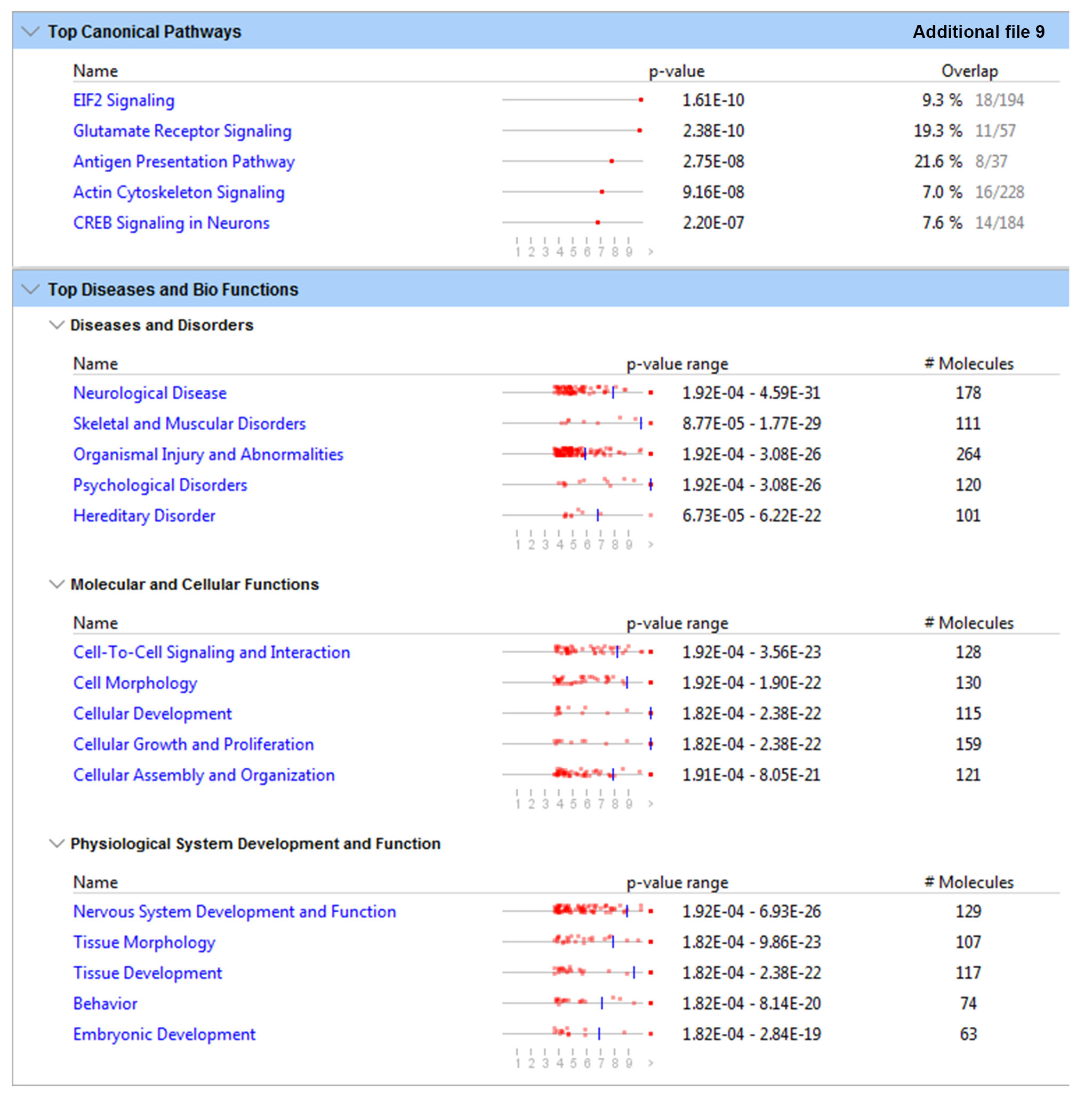

Supplement: Supplementary file 9 — Affected top canonical pathways, diseases and disorders, molecular and cellular functions, and physiological system development and function after chronic T. gondii infection according to Ingenuity Pathway Analysis (IPA™). Ingenuity Pathway Analysis (IPA™) of our data set according to Additional file 3 reveals a large number of canonical pathways, diseases and disorders, molecular and cellular functions, and physiological system development and function represented in our data with high significance. These findings agree with former observations related to T. gondii infection. (TIF 3320 kb) [file 12974_2018_1242_MOESM9_ESM.tif]
