# Supplementary material for: Chronic Toxoplasma infection is associated with distinct alterations in the synaptic protein composition
Source: J Neuroinflammation. 2018 Aug 1;15:216. doi: 10.1186/s12974-018-1242-1 (PMC6090988; doi:10.1186/s12974-018-1242-1)
Supplement: Supplementary file 5 — Proteomic analyses revealed robust quantification of protein abundances in isolated synaptosomes. Venn diagram showing all proteins found in four separate sample pairs (1–4). Numbers (dark gray) indicate the number of proteins found in corresponding sample pairs. Total numbers of found proteins in each single sample pair are given in parentheses. (PDF 2370 kb) [file 12974_2018_1242_MOESM5_ESM.pdf]

Additional file 5

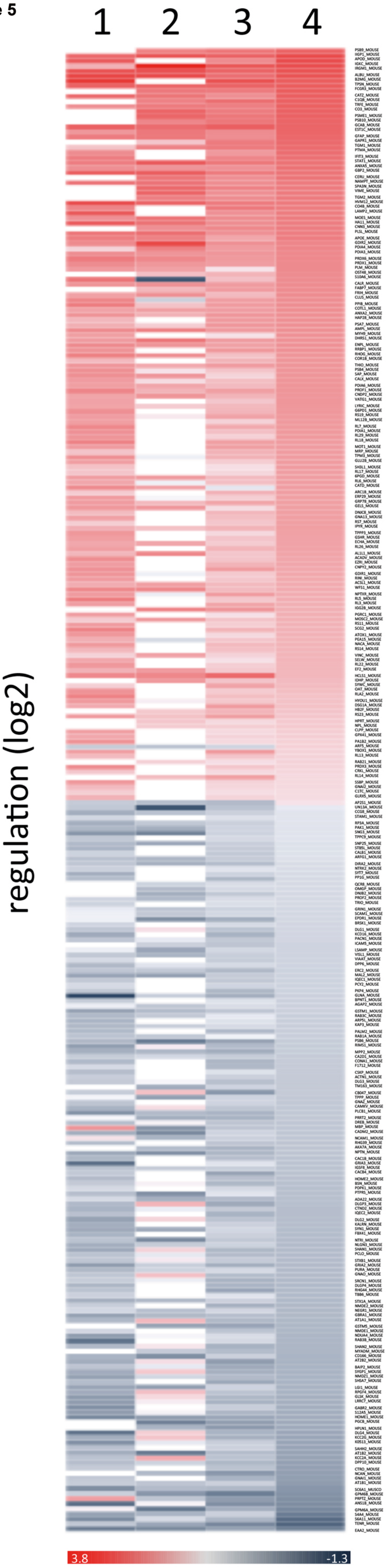

Proteomic analysis of synaptosomes from *Toxoplasma gondii* infected mice. Heatmaps showing relative protein abundances (log2) of all 292 significantly regulated proteins in synaptosomes after *Toxoplasma gondii* infection in four separate sample pairs. Colour codes are indicated.
