# Supplementary material for: Chronic Toxoplasma infection is associated with distinct alterations in the synaptic protein composition
Source: J Neuroinflammation. 2018 Aug 1;15:216. doi: 10.1186/s12974-018-1242-1 (PMC6090988; doi:10.1186/s12974-018-1242-1)
Supplement: Supplementary file 6 — Glutamate receptor signaling pathway according to IPA™. Symbols are explained in a table (part B). Filled symbols represent proteins found to be altered in synaptosomes according to our MS data, green indicates reduced levels, and red notifies increased levels compared to controls. (PDF 1650 kb) [file 12974_2018_1242_MOESM6_ESM.pdf]

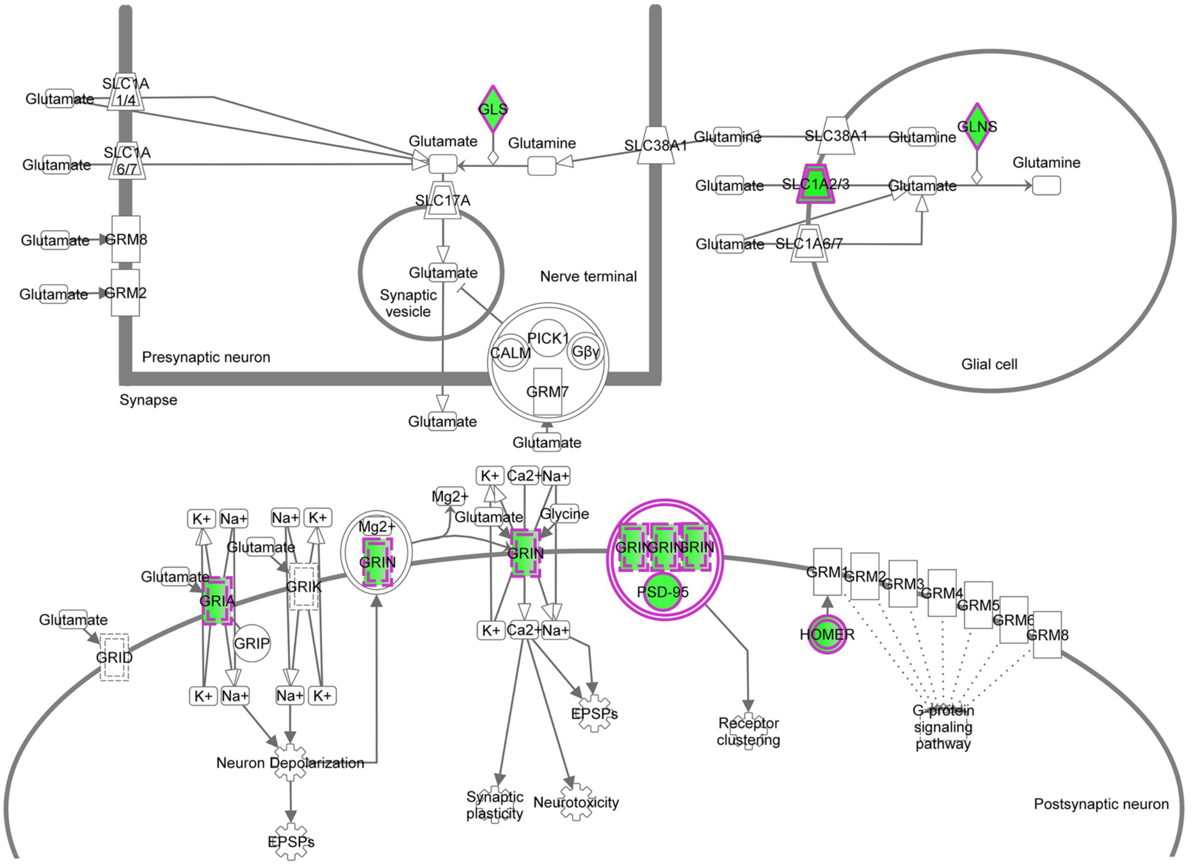

Additional file 6

Glutamate receptor signaling pathway according to IPA®

Symbols are explained in a table (Part B). Filled symbols represent proteins found to be altered in synaptosomes according to our MS data, green indicates reduced levels and red notifies increased levels compared to controls. The networks and functional analyses were generated through the use of QIAGEN's Ingenuity Pathway Analysis (IPA®, QIAGEN Redwood City, www.qiagen.com/ingenuity).

Symbol	Synonym(s)	Location	Family
Ca2+	calcium ion (Ca2+)	Other	chemical - endogenous mammalian
CALM	CALM, Cam	Cytoplasm	group
Calmodulin-G protein ßγ-Grm7-Pick1	Calmodulin-G protein β γ-Grm7-Pick1	Plasma Membrane	complex
EPSPs		Other	function
Gβγ	G-protein β/γ,	Cytoplasm	complex
GLNS	Glutamine Synthetase	Cytoplasm	enzyme
GLS	glutaminase	Cytoplasm	enzyme
Glutamate	L-glutamate	Other	chemical - endogenous mammalian
Glutamine	L-glutamine	Other	chemical - endogenous mammalian
Glycine	glycine	Other	chemical - endogenous mammalian
GRIA	AMPAR, GluA, iGluR	Plasma Membrane	group
GRID		Plasma Membrane	group
GRIK	kainate glutamate receptor	Plasma Membrane	group
GRIN	GRIN, NMDAR, NMDARs	Plasma Membrane	complex
Grin-Grin-Grin-Dlg4		Plasma Membrane	complex
Grin-Mg2+	Grin-Mg2+	Plasma Membrane	complex
GRIP	glutamate receptor interacting protein 1, GRIP	Plasma Membrane	transcription regulator
GRM1	Metabotropic glutamate receptor 1a	Plasma Membrane	G-protein coupled receptor
GRM2	Metabotropic glutamate receptor 2	Plasma Membrane	G-protein coupled receptor
GRM3	Metabotropic glutamate receptor 3	Plasma Membrane	G-protein coupled receptor
GRM4	Metabotropic glutamate receptor 4	Plasma Membrane	G-protein coupled receptor
GRM5	Metabotropic glutamate receptor 5a	Plasma Membrane	G-protein coupled receptor
GRM6	Metabotropic glutamate receptor 6	Plasma Membrane	G-protein coupled receptor
GRM7	Metabotropic glutamate receptor 7	Plasma Membrane	G-protein coupled receptor
GRM8	Metabotropic glutamate receptor 8	Plasma Membrane	G-protein coupled receptor
HOMER		Cytoplasm	group
K+	potassium ion (K+)	Other	chemical - endogenous mammalian
Mg2+	magnesium ion (Mg2+)	Other	chemical - endogenous mammalian
Na+	sodium cation (Na+)	Other	chemical - endogenous mammalian
Neuron Depolarization		Other	function
Neurotoxicity		Other	function
PICK1	protein interacting with C kinase 1	Cytoplasm	enzyme
PSD-95	PSD-95	Plasma Membrane	kinase
Receptorclustering		Other	function
SLC17A		Plasma Membrane	group
SLC1A1/4		Plasma Membrane	group
SLC1A2/3		Plasma Membrane	group
SLC1A6/7		Plasma Membrane	group
SLC38A1	solute carrier family 38 member 1	Plasma Membrane	transporter
Synapticplasticity		Other	function
