# Supplementary material for: Chronic Toxoplasma infection is associated with distinct alterations in the synaptic protein composition
Source: J Neuroinflammation. 2018 Aug 1;15:216. doi: 10.1186/s12974-018-1242-1 (PMC6090988; doi:10.1186/s12974-018-1242-1)
Supplement: Supplementary file 7 — GABA receptor signaling pathway according to IPA™. Symbols are explained in a table (part B). Filled symbols represent proteins found to be altered in synaptosomes according to our MS data, green indicates reduced levels, and red notifies increased levels compared to controls. (PDF 1430 kb) [file 12974_2018_1242_MOESM7_ESM.pdf]

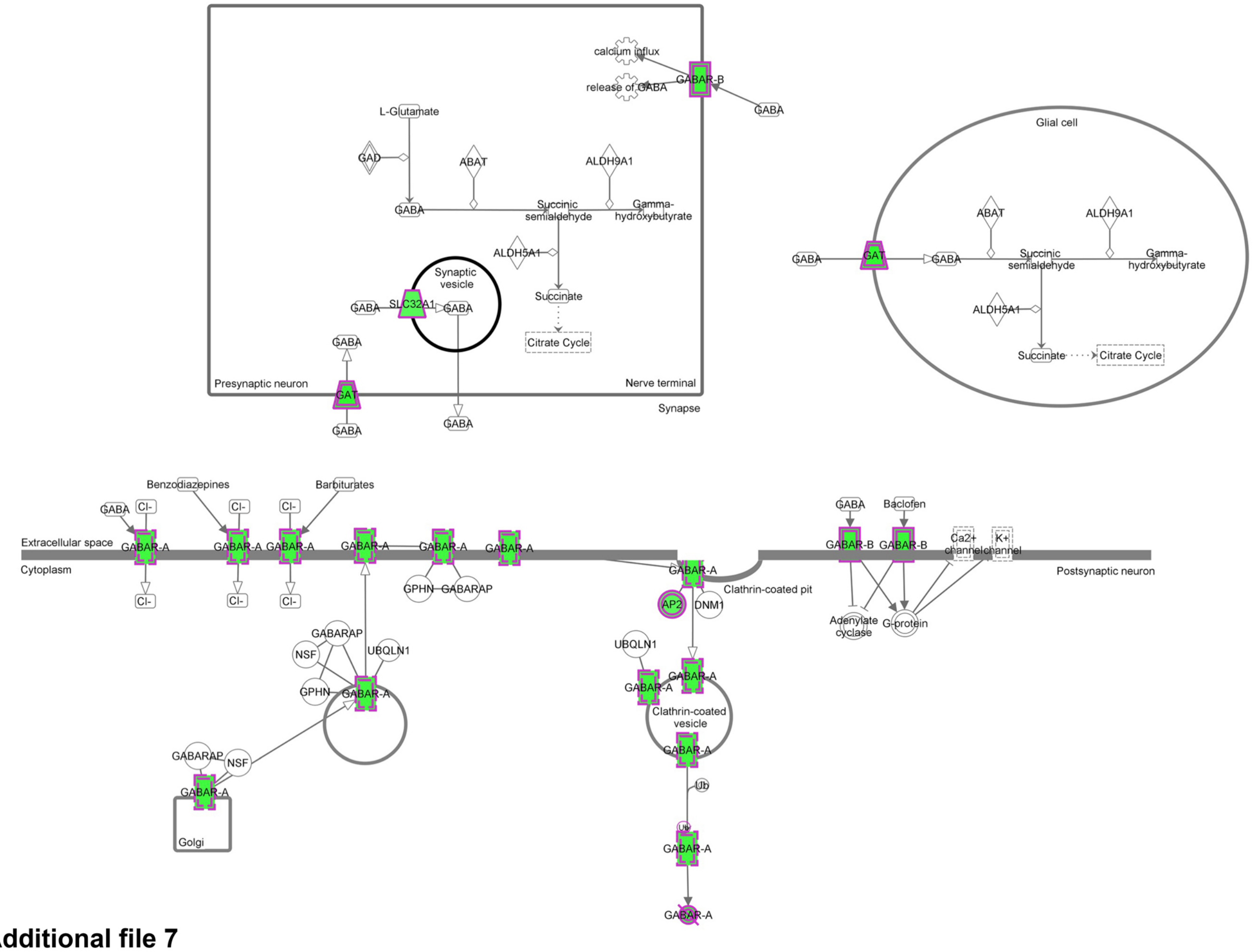

Additional file 7

GABA receptor signaling pathway according to IPA®

Symbols are explained in a table (Part B). Filled symbols represent proteins found to be altered in synaptosomes according to our MS data, green indicates reduced levels and red notifies increased levels compared to controls. The networks and functional analyses were generated through the use of QIAGEN's Ingenuity Pathway Analysis (IPA®, QIAGEN Redwood City, www.qiagen.com/ingenuity)

Symbol	Synonym(s)	Location	Family
ABAT	4-aminobutyrate aminotransferase	Cytoplasm	enzyme
Adenylatecyclase	3',5'-cyclic AMP synthetase	Cytoplasm	group
ALDH5A1	aldehyde dehydrogenase 5 family member A1	Cytoplasm	enzyme
ALDH9A1	aldehyde dehydrogenase 9 family member A1	Cytoplasm	enzyme
AP2	AP-2, Clathrin adaptor AP2	Cytoplasm	complex
Baclofen	Apo-Baclofen, Baclon	Other	chemical drug
Barbiturates		Other	chemical - other
Benzodiazepines		Other	chemical drug
Ca2+channel	Voltage-Gated Ca2+ Channel	Plasma Membrane	complex
calcium influx		Other	function
Cl-	chloride ion (Cl-)	Other	chemical - endogenous mammalian
DNM1	dynamin 1	Cytoplasm	enzyme
G-protein	G-protein α-β-γ	Cytoplasm	complex
GABA	γ-aminobutyric acid	Other	chemical - endogenous mammalian
GABAR-A	GABA type A receptor	Plasma Membrane	complex
GABAR-B	GABA type B receptor	Plasma Membrane	group
GABARAP	GABA type A receptor-associated protein	Cytoplasm	transporter
GAD	γ-glutamate decarboxylase, Gad65/67	Other	group
Gamma-hydroxybutyrate	γ-Hydroxybutyric acid	Other	chemical drug
GAT	GABA transporter	Other	group
GPHN	gephyrin	Plasma Membrane	enzyme
K+channel	potassium channel	Plasma Membrane	group
L-Glutamate	L-glutamic acid	Other	chemical - endogenous mammalian
NSF	N-ethylmaleimide sensitive factor / vesicle fusing ATPase,	Cytoplasm	transporter
release of GABA		Other	function
SLC32A1	solute carrier family 32 (GABA vesicular transporter) member 1	Plasma Membrane	transporter
Succinate	succinate	Other	chemical - endogenous mammalian
Succinic semialdehyde	γ-oxybutyric acid	Other	chemical - endogenous mammalian
Ub	Polyubiquitin, Ub	Cytoplasm	group
UBQLN1	ubiquilin 1	Cytoplasm	other
