# Supplementary material for: Chronic Toxoplasma infection is associated with distinct alterations in the synaptic protein composition
Source: J Neuroinflammation. 2018 Aug 1;15:216. doi: 10.1186/s12974-018-1242-1 (PMC6090988; doi:10.1186/s12974-018-1242-1)
Supplement: Supplementary file 10 — Antigen presentation pathway according to IPA™. Symbols are explained in a table (part B). Filled symbols represent proteins found to be altered in synaptosomes according to MS data, green indicates reduced levels, and red notifies increased levels compared to controls. (PDF 1690 kb) [file 12974_2018_1242_MOESM10_ESM.pdf]

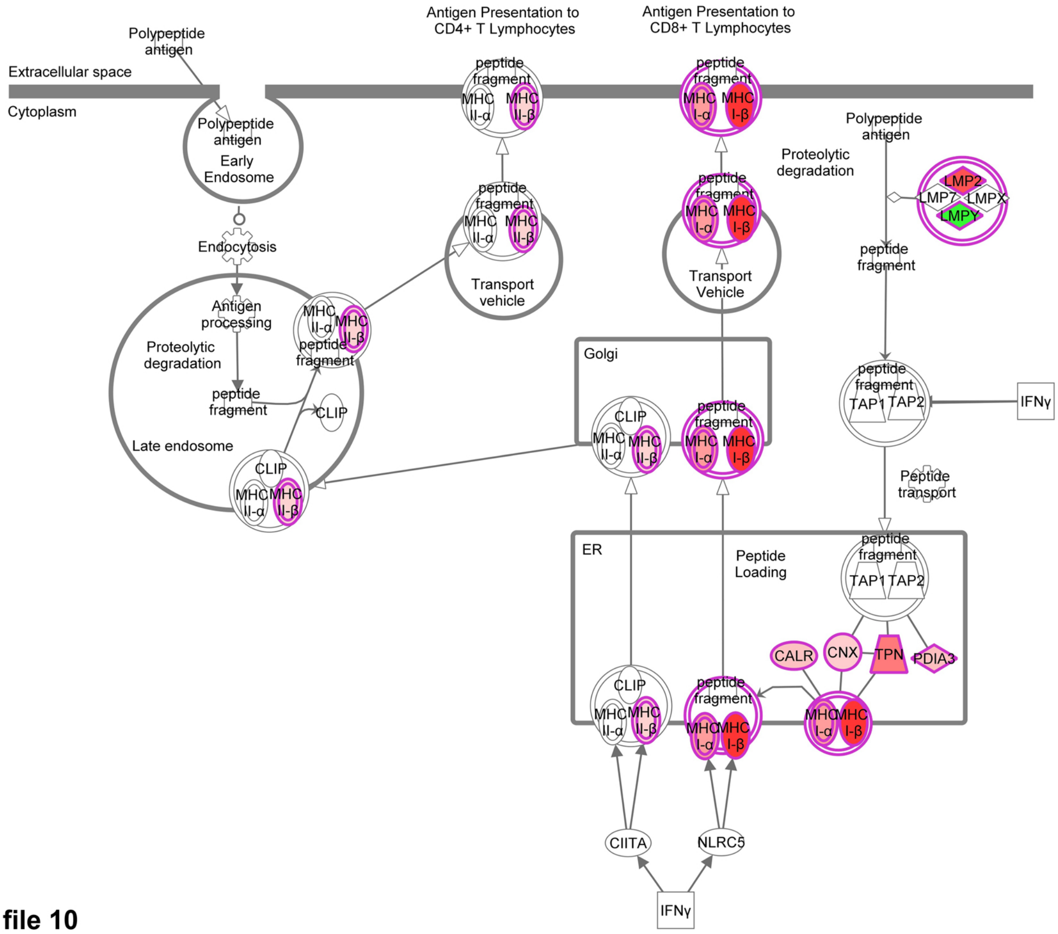

Additional file 10

Antigen presentation pathway according to IPA®

Symbols are explained in a table (Part B). Filled symbols represent proteins found to be altered in synaptosomes according to our MS data, green indicates reduced levels and red notifies increased levels compared to controls. The networks and functional analyses were generated through the use of QIAGEN's Ingenuity Pathway Analysis (IPA®, QIAGEN Redwood City, www.qiagen.com/ingenuity).

Symbol	Synonym(s)	Location	Family
Antigenprocessing		Other	function
B2m-Mhc1a-peptide fragment	B2m-Mhc1a-peptide fragment	Plasma Membrane	complex
CALR	Calreticulin	Cytoplasm	transcription regulator
CD74-Mhc2a-Mhc2b		Cytoplasm	complex
CIITA	Class II major histocompatibility complex transactivator	Nucleus	transcription regulator
CLIP	CD74 antigen (H-2 class II histocompatibility antigen gamma chain)	Plasma Membrane	transmembrane receptor
CNX	Calnexin	Cytoplasm	other
Endocytosis		Other	function
IFNγ	Interferon gamma (Interferon γ)	Extracellular Space	cytokine
LMP2	Large multifunctional protease 2 (LMP2, proteasome subunit beta 9)	Cytoplasm	peptidase
LMP7	Large multifunctional protease-7 (LMP7, proteasome subunit beta 8)	Cytoplasm	peptidase
LMPX	Proteasome subunit beta 5 (LMPX, proteasome 20S X)	Cytoplasm	peptidase
LMPY	Proteasome subunit beta 6 (LMPY, proteasome subunit delta)	Nucleus	peptidase
MHC I-α	MHC class I alpha	Plasma Membrane	group
MHC I-β	Beta-2-microglobulin (MHC class I beta, β2M)	Plasma Membrane	transmembrane receptor
MHC II-α	MHC II alpha chain	Plasma Membrane	group
MHC II-β	MHC II beta chain	Plasma Membrane	group
Mhc2a-Mhc2b-peptide fragment	Mhc2a-Mhc2b-peptide fragment	Plasma Membrane	complex
NLRC5	NLR family CARD domain containing 5	Cytoplasm	transcription regulator
PDIA3	Protein disulfide-isomerase A3 (GRP58)	Cytoplasm	peptidase
Peptide transport		Other	function
peptide-Tap1-Tap2		Cytoplasm	complex
Psmb5-Psmb6-Psmb8-Psmb9		Cytoplasm	complex
TAP1	Antigen peptide transporter 1 (APT1, peptide transporter TAP1)	Cytoplasm	transporter
TAP2	Antigen peptide transporter 2 (APT2, peptide transporter TAP2)	Cytoplasm	transporter
TPN	TAP binding protein (Tapasin)	Cytoplasm	transporter
