# Supplementary material for: Chronic Toxoplasma infection is associated with distinct alterations in the synaptic protein composition
Source: J Neuroinflammation. 2018 Aug 1;15:216. doi: 10.1186/s12974-018-1242-1 (PMC6090988; doi:10.1186/s12974-018-1242-1)
Supplement: Supplementary file 11 — Lipid antigen presentation pathway according to IPA™. Symbols are explained in a table (part B). Filled symbols represent proteins found to be altered in synaptosomes according to MS data, green indicates reduced levels, and red notifies increased levels compared to controls. (PDF 1830 kb) [file 12974_2018_1242_MOESM11_ESM.pdf]

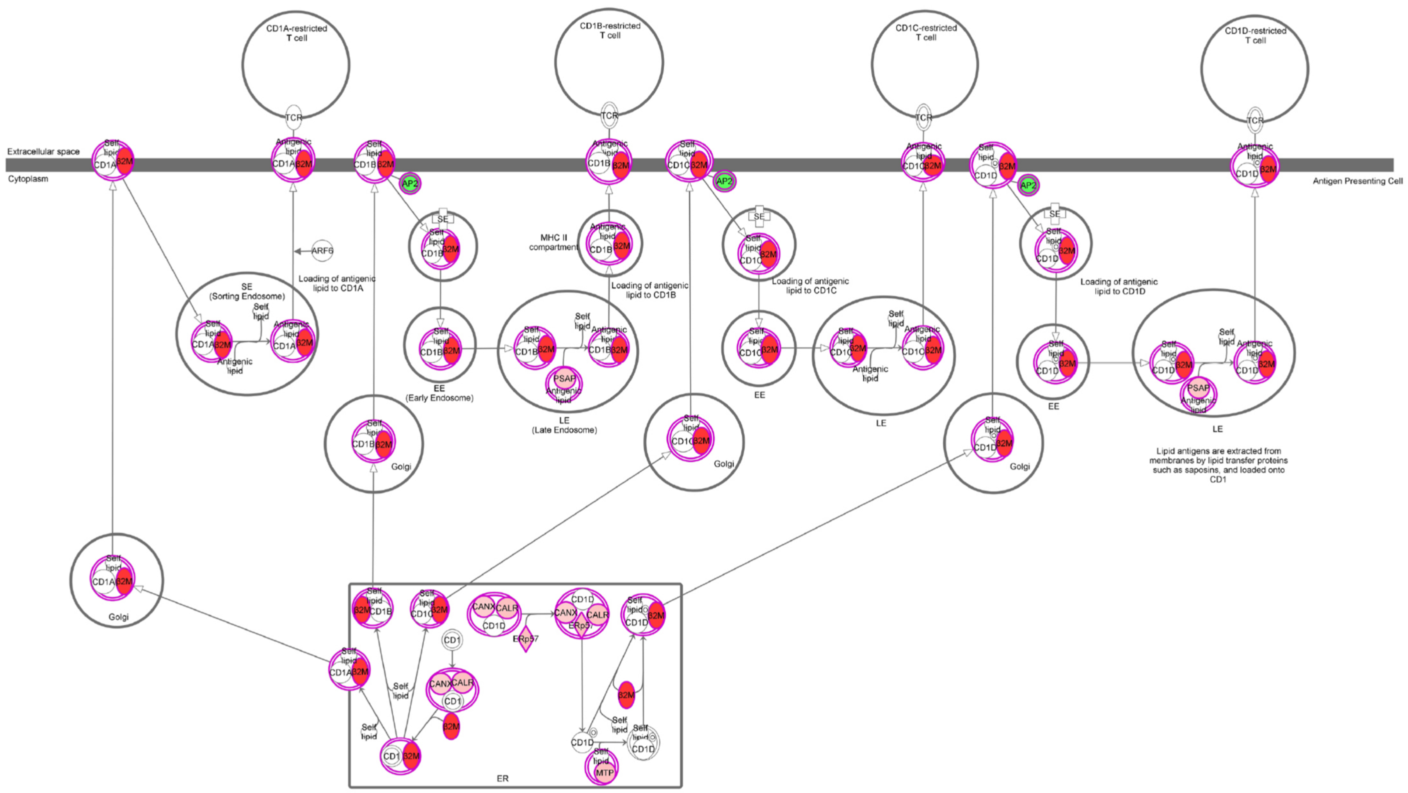

Additional file 11

Lipid antigen presentation pathway according to IPA®

Symbols are explained in a table (Part B). Filled symbols represent proteins found to be altered in synaptosomes according to our MS data, green indicates reduced levels and red notifies increased levels compared to controls. The networks and functional analyses were generated through the use of QIAGEN's Ingenuity Pathway Analysis (IPA®, QIAGEN Redwood City, www.qiagen.com/ingenuity).

Symbol	Synonym(s)	Location	Family
β2M	Beta-2-microglobulin (MHC class I beta, β2M)	Plasma Membrane	transmembrane receptor
Antigenic lipid-CD1A-β2M	Antigenic lipid-CD1A-beta2M	Plasma Membrane	complex
Antigenic lipid-CD1B-β2M	Antigenic lipid-CD1B-beta2M	Plasma Membrane	complex
Antigenic lipid-CD1C-β2M	Antigenic lipid-CD1C-beta2M	Plasma Membrane	complex
Antigenic lipid-CD1D-β2M	Antigenic lipid-CD1D-beta2M	Plasma Membrane	complex
AP2	Clathrin adaptor AP2	Cytoplasm	complex
ARF6	ADP-ribosylation factor 6	Plasma Membrane	transporter
CALR	Calreticulin	Cytoplasm	transcription regulator
CANX	Calnexin	Cytoplasm	other
CANX-CALR-CD1		Cytoplasm	complex
CD1	CD1-Family of T-cell surface glycoprotein	Plasma Membrane	group
CD1-β2MComplex	CD1-beta2M Complex	Cytoplasm	complex
CD1A	T-cell surface glycoprotein CD1a	Plasma Membrane	other
CD1B	T-cell surface glycoprotein CD1b	Plasma Membrane	other
CD1C	T-cell surface glycoprotein CD1c	Plasma Membrane	other
CD1D	T-cell surface glycoprotein CD1d	Plasma Membrane	other
CD1D-CANX-CALR		Cytoplasm	complex
CD1D-CANX-CALR-ERp57		Cytoplasm	complex
ERp57	Protein disulfide-isomerase A3 (PDIA3, GRP58)	Cytoplasm	peptidase
PSAP	Prosaposin (Sphingolipid activator protein)	Extracellular Space	other
PSAP-Antigenic lipid		Cytoplasm	complex
Self lipid-CD1A-β2M	Self lipid-CD1A-beta2M	Cytoplasm	complex
Self lipid-CD1B-β2M	Self lipid-CD1B-beta2M	Cytoplasm	complex
Self lipid-CD1C-β2M	Self lipid-CD1C-beta2M	Cytoplasm	complex
Self lipid-CD1D	Self lipid-CD1D	Cytoplasm	complex
Self lipid-CD1D-β2M	Self lipid-CD1D-beta2M	Cytoplasm	complex
TCR	T-cell receptor	Plasma Membrane	complex
